# Synthesis of Degraded Limonoid Analogs as New Antibacterial Scaffolds against *Staphylococcus aureus*

**DOI:** 10.3390/antibiotics9080488

**Published:** 2020-08-06

**Authors:** Marta Ferrera-Suanzes, Victoria Prieto, Antonio J. Medina-Olivera, José Manuel Botubol-Ares, Fátima Galán-Sánchez, Manuel A. Rodríguez-Iglesias, Rosario Hernández-Galán, María Jesús Durán-Peña

**Affiliations:** 1 Department of Organic Chemistry, Faculty of Sciences, Campus Universitario Río San Pedro s/n, Torre Sur, 4^a^; planta, University of Cádiz, 11510 Puerto Real, 11009 Cádiz, Spain; marta.ferrerasuanzes@alum.uca.es (M.F.-S.); antoniojesus.medinaolivera@alum.uca.es (A.J.M.-O.); josemanuel.botubol@uca.es (J.M.B.-A.); rosario.hernandez@uca.es (R.H.-G.); 2 Department of Biomedicine, Biotechnology and Public Health, Hospital Puerta del Mar, University of Cádiz, 11009 Cádiz, Spain; vivprieto@gmail.com (V.P.); fatima.galan@uca.es (F.G.-S.); manuel.rodrigueziglesias@uca.es (M.A.R.-I.); 3 Instituto de investigación e Innovación Biomédica de Cádiz (INIBICA), 11009 Cádiz, Spain

**Keywords:** degraded limonoids, drug design, *S. aureus*, MRSA, drug-like properties

## Abstract

*Staphylococcus aureus* and methicillin-resistant *Staphylococcus aureus* (MRSA) have become serious infections in humans and ruminants. *S. aureus* strains are showing rapid changes to develop resistance in traditional antibiotic-containing systems. In the continuous fierce fight against the emergent multi-drug resistant bacterial strains, straightforward and scalable synthetic procedures to produce new active molecules are in demand. Analysis of molecular properties points to degraded limonoids as promising candidates. In this article, we report a simple synthetic approach to obtain degraded limonoid analogs as scaffolds for new antibacterial molecules. The minimum inhibitory concentrations against *S. aureus* were evaluated for the stereoisomer mixtures by the broth microdilution method. Analysis of results showed that the acetylated derivatives were the most active of them all.

## 1. Introduction

Multidrug resistance (MDR) is one of the emergent problems in healthcare in recent times due to the loss of effective activity of some drugs against multiresistant bacteria [1]. Infections caused by multidrug-resistant bacteria such as methicillin-resistant *Staphylococcus aureus* (MRSA), linezolid-resistant *Staphylococcus* spp. or vancomycin-resistant *Enterococcus faecium* represent a growing worldwide issue [2,3]. Developing new antimicrobial agents is too slow versus the urgent need to combat bacterial pathogens [4]. To achieve a meaningful control of these multiresistant strains, that become serious infections in humans and ruminants [5,6], new approaches to obtain active molecules are in demand.

**Figure 1:**
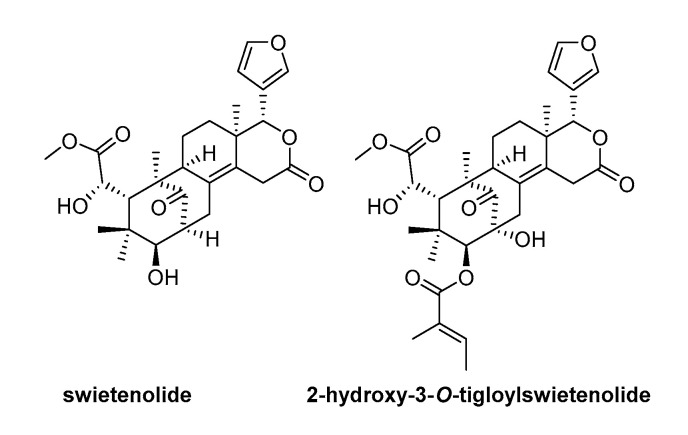
Structure of natural limonoids with antibacterial activity against multidrug resistance (MDR) bacterial strains.

Due to the diverse biological activities showed by natural products (NPs), they comprise a rich resource for studies about structural complexity and functional group diversity [7,8]. For this reason, NPs are still a potent source of inspiration in drug discovery [9,10,11,12]. Among NPs, secondary metabolites from plants with terpene structures have proven numerous clinical trials and drugs [13]. Limonoids are tetranortriterpenoids isolated from Meliaceae and Rutaceae plants [14]. In literature, two limonoids are described to exhibit multiple-drug resistance activity against eight MDR bacterial strains including *S. aureus* strains (Figure 1, **swietenolide** and **2-hydroxy-3-*O*-tigloylswietenolide**) [15].

Unfortunately, the availability of limonoids from natural sources is limited and their total syntheses are a challenge because they usually require a considerable number of reaction steps [16,17].

When designing new antibacterial drugs some aspects should be kept in mind such as avoiding too long multi-step total synthesis or semi-synthesis starting from NPs averting biodisponibility problems to contribute to the scalability of synthetically-accessible drug-like molecules, as well as foreseeing good drug-likeness molecular properties. Currently, some sectors of the pharmaceutical industry continue producing compounds that have suboptimal physicochemical profiles [18], decreasing the likelihood of success in terms of the development of these molecules as drugs [18,19]. Recent studies have been described to predict the bioavailability of small molecules about physicochemical properties involve in the drug-likeness characteristics [20,21]. The well-known Lipinski’s rules—also named as the “rule of five”—has gained acceptance as an approach to design, discover, and develop new bioactive molecules [19]. Nevertheless, these properties and others have been analyzed or matched up in other approaches, which provide required parameters for drugability. The application of all these guidelines linked to the concept of drug-likeness could contribute to drawing up a preliminary analysis of the molecules to be considered as drug-like compounds based on these predictions.

## 2. Results

### 2.1. Design of Model Molecules Based on Degraded Limonoid Skeleton and Molecular Properties Prediction

Phytochemical studies have revealed a great variety of pharmacological activities [22,23,24] as well as health-promoting and disease-preventing properties exhibited by limonoids [25,26].

For designing new antibacterial compounds based on limonoid structures, we first evaluated the molecular properties of **swietenolide** and **2-hydroxy-3-*O*-tigloylswietenolide.** The freely available ADMETlab database website was employed to predict the administration, distribution, metabolism, excretion, and toxicity properties (ADMET) from these chemical structures [27,28]. We analyzed a set of physicochemical descriptors for **swietenolide** and **2-hydroxy-3-*O*-tigloylswietenolide** according to Lipinski’s rules [29,30], Ghose’s rules [31], Veber’s rules [32], Varma’s rules [33], and Oprea’s rules [34] that attempt to make predictions of drug-likeness (Table S1). With this aim, the range of drug-like parameters according to 11 molecular properties were studied (Table 1).

As opposed to these complex biologically-active limonoids, their simpler related degraded limonoids have also shown promising biological effects such as anticancer [35], antifungal [36], neuroprotective [37], anti-trypanosomal [38], and antibacterial activities [39]. 

**Figure 2:**
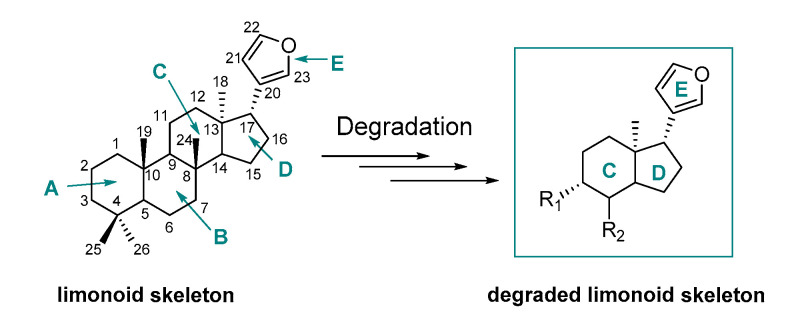
Limonoid and degraded limonoid skeleta.

A common feature in the different limonoid families and the degraded limonoids is the main motif *δ*-lactone with a 3-furyl substituent (Figure 2).

Based on the purpose of designing new active and non-complex molecules—avoiding unnecessary ornaments in the structures—in this study we proposed to use a degraded limonoid skeleton as a scaffold for the synthesis of new antibacterial agents. 

**Figure 3:**
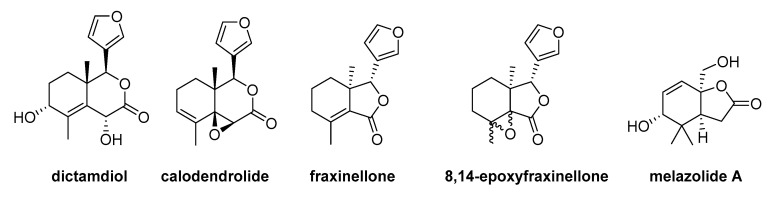
Chemical structures of some naturally-occurring degraded limonoids.

Theoretical molecular properties were calculated to predict if some representative degraded limonoids (Table S2) could match the required parameters for drugability (Table 2). Some of these metabolites show relevant pharmacological or agrochemical effects (Figure 3) [40,41,42,43].

**Figure 4:**
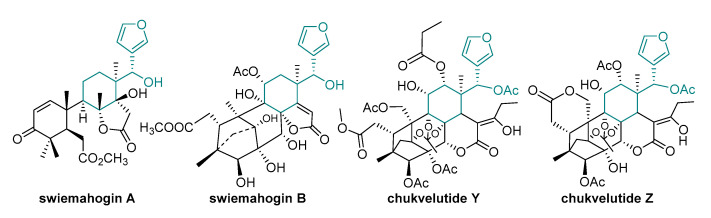
Chemical structures of some phragmalin-type limonoids.

On the other hand, some natural phragmalin-type limonoids possess the D-ring *δ*-lactone demolished such as swiemahogins A and B [44], or chukvelutides [45] (Figure 4).

**Scheme 1:**
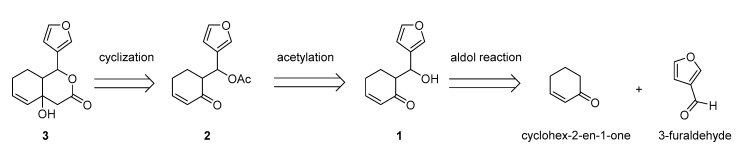
Retrosynthetic route for the synthesis of simplified model molecules **1**–**3**.

With all these above considerations, the degraded limonoid analogs **1**–**3** were proposed to be synthetized as model molecules (Scheme 1). The previous phragmalin-type limonoids share a common pattern with **1**−hydroxyl group on the carbon connected to furanyl group−or **2**−acetyl group on the carbon connected to furanyl group−, respectively. We proposed the synthesis of compounds **1**–**3** to evaluate if these modifications in the D ring could induce different results.

Physicochemical descriptors for the model compounds **1**–**3** were also calculated (Table S2) and they were compared with those belonging to the MDR limonoids **swietenolide** and its derivative **2-hydroxy-3-*O*-tigloylswietenolide**.

### 2.2. Synthesis of the Degraded Limonoid Analogs 1–3 and Chemical Characterization

**Scheme 2:**
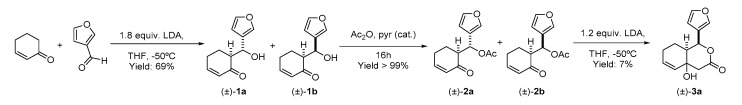
Synthesis of model molecules **1**–**3**.

Following the strategy used for the synthesis of some limonoids described in the literature [46,47], treatment of cyclohex-2-en-1-one with the sterically hindered base lithium diisopropylamide (LDA) in THF at −50 °C and subsequent condensation of its lithium enolate with the commercially available 3-furaldehyde afforded the mixture of stereoisomers (±)-**1a** and (±)-**1b** in 69% yield. Then, these *β*-hydroxy ketones (±)-**1a** and (±)-**1b** were acetylated under standard conditions—acetic anhydride and a catalytic amount of pyridine—to give quantitatively the compounds (±)-**2a** and (±)-**2b**. At this point, the mixture of (±)-**2a** and (±)-**2b** was subjected to an intramolecular aldol reaction employing LDA in THF at −50 °C to afford the *δ*-valerolactone (±)-**3a** in 7% yield as an isolated product. It is remarkable that in this cyclization reaction one diastereoisomer out of four possible could be only purified by chromatography column using mixtures of hexane:ethyl acetate in different proportions possibly due to the reaction crude was difficult to solubilize (Scheme 2).

Compounds **1** and **2** have two stereocenters. Therefore, these mixtures are made up of four isomers, which are two diastereoisomers and two enantiomer pairs. The structures of the synthesized compounds **1**–**3** were characterized by mass spectrometry, ^1^H NMR, ^13^C NMR, and Fourier transform IR (FT-IR) spectroscopy.

**Figure 5:**
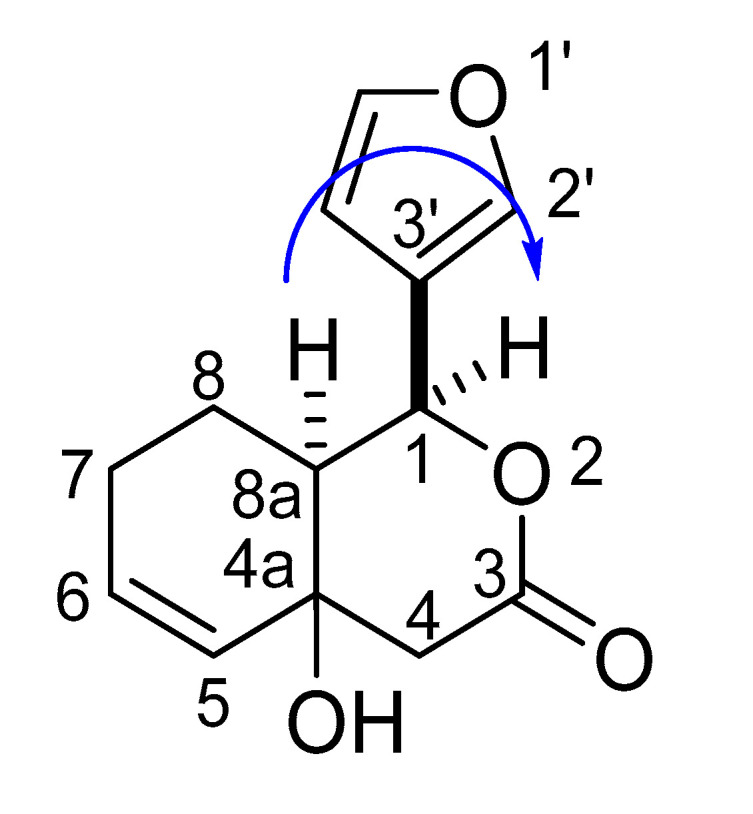
Key n.O.e. correlations for (±)-**3a**.

Furthermore, in the NMR spectra of compound (±)-**3a** there were nuclear Overhauser effect (n.O.e.) correlations between H-8a and H-1 that supported the relative configuration of the ring as 1S*,8aS* (Figure 5).

### 2.3. Antimicrobial Activity of the Degraded Limonoid Analogs 1–3

The minimal inhibitory concentration (MIC) of degraded limonoid analogs **1**–**3** was determined on 96 well culture plates for reference strain *S. aureus* ATCC 25923 and a set of methicillin-resistant *S. aureus* clinical isolates by using microdilution assay and a microorganism suspension (Table 3).

Then, we evaluated the behavior of stereoisomer mixture **2** ((±)-**2a** and (±)-**2b**) against other Gram-positive bacteria (Table 4). 

### 2.4. Time–Kill Curves

**Figure 6:**
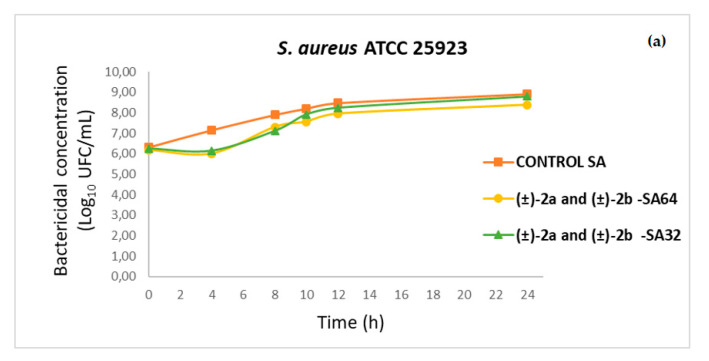

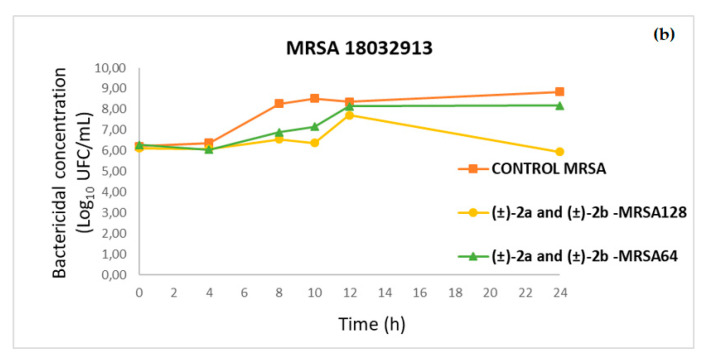
(**a**) Time-kill curve of *S. aureus* ATCC 25923; (**b**) Time-kill curve of MRSA 18032913.

Using time course assays, time-kill curve assays were performed and we examined the bactericidal activity of the stereoisomers 2 ((±)-2a and (±)-2-b) against *S. aureus* ATCC 25923 and MRSA 18032913 (Figure 6). 

Stereoisomers 2 ((±)-2a and (±)-2b) presented bactericidal effects against *S. aureus* at 64 and 32 mg/L after 4 h. These effects are decreasing whenever the time is increasing. These reductions did not persist noteworthy at 24 h.

In the same way, 2 presented bactericidal effects against MRSA 18032913 showing a significant decrease in the first 8–10 h at both concentrations tested. After 24 h, the difference in bactericidal concentration became clearer than in previous measurements at 128 mg/L whereas at 64 mg/L the inhibition is slightly lower than the control measurement.

### 2.5. Synthesis and Antimicrobial Activity of More Lipophilic Degraded-Limonoid Analogs 4–6 and Chemical Characterization

**Scheme 3:**
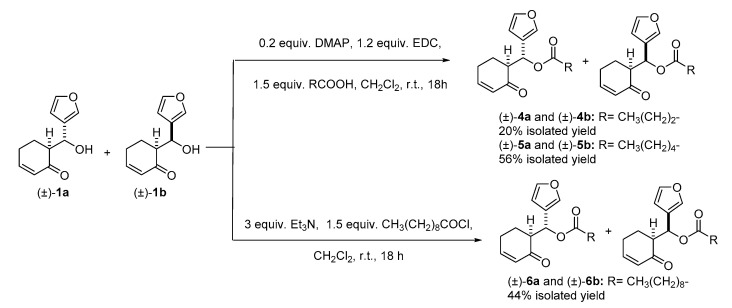
Synthesis of model molecules 4–6.

The procedures carried out to synthetize the more lipophilic derivatives 4–6 by Steglich esterification and *O*-acylation reactions are shown on Scheme 3.

Further purification by high-performance liquid chromatography (HPLC) or chromatography column afforded compounds 4–6 as single diastereomers and the two series were labeled as “a” for the less polar enantiomer pair and ‘b’ for more polar enantiomer pair according to the TLC plates using 25% hexane:ethyl acetate as eluent. The structures of the diastereoisomers of furan-3-yl-(2-oxocyclohex-3-en-1-yl)methyl butyrate ((±)-4a and (±)-4b), furan-3-yl-(2-oxocyclohex-3-en-1-yl)methyl hexanoate ((±)-5a, (±)-5b), and furan-3-yl-(2-oxocyclohex-3-en-1-yl)methyl decanoate ((±)-6a and (±)-6b) were separately characterized by mass spectrometry, ^1^H NMR, ^13^C NMR and FT-IR spectroscopy.

Diastereoisomers (±)-4a-(±)-6a and (±)-4b-(±)-6b were tested against *S. aureus* ATCC 25923. The MIC_50_ values are presented in Table 5. These data showed that these compounds are all less active than mixture (±)-2a and (±)-2b (MIC_50_ = 16 mg/L).

## 3. Discussion

Oral bioavailability is correlated to Lipinski’s rules in terms of the number of hydrogen bond donors (≤5), the number of hydrogen bond acceptors (≤10), molecular weight (≤500 g/mol), and log P (≤5). According to these data, **swietenolide** falls within the typical Lipinski’s rules values giving 100% matches (Table S1), whereas its derivative **2-hydroxy-3-*O*-tigloylswietenolide** only matches in 75% due to the molecular weight of this natural product is higher than 500 g/mol (Table S1). Unexpectedly, these naturally-occurring limonoids do not perfectly conform to all the remaining rules except in Veber’s rules for **swietenolide**. **Swietenolide** has four rotatable bonds (less than six) reducing it matches coincidence with Oprea´s rules (Table S1). **2-Hydroxy-3-*O*-tigloylswietenolide** does not fit in Ghose´s rules due to the molar refractivity, the total number of atoms and molecular weight are all higher than the typical values for drugability (Table S1). The total polar surface area (TPSA)—a parameter included in both Veber and Varma´s rules—is used as a tool to estimate the transport pathway of the particular type of drug [48]. The TPSA value for **2-hydroxy-3-*O*-tigloylswietenolide** is 149.57 Å, slightly higher than 140 Å—upper in Veber´s rules—and much higher than Varma´s predictions (TPSA ≤120 Å) (Table S1). According to Varma´s rules, this molecule is in concordance with just 40% with these rules (Table S1).

Despite the promising bacterial inhibition showed by **swietenolide** and its derivative, it would be desirable to improve the molecular properties as a key aspect to design new antibacterial compounds. Getting better molecular properties might increase the likelihood of success when producing a drug with good pharmacokinetic and pharmacodynamics parameters. Calculations of the physicochemical descriptors included in the mentioned rules for naturally degraded limonoids—**dictamdiol**, **calodendrolide**, **fraxinellone**, **8,14-epoxyfraxinellone**, and **melazolide A**—resulted in excellent properties to be considered as interesting biologically-active structures (100% matches in Lipinski, Ghose, Veber, and Varma’s rules, although lower percentage matches are predicted for Oprea´s rules) (Table S2).

Based on the favorable properties calculated for degraded limonoids and to synthesize model molecules characterized by great structural simplicity, we chose degraded limonoids for the design of new antibiotics with simple structures, easy to synthesize and that would preserve those properties. Therefore, after a careful analysis of their drug-like properties, the synthesis of the degraded limonoid analogs **1**–**3** was proposed (Table S2). It was expected to anticipate good biological properties for **1**–**3** because most of the properties included in the rules for prediction of drug-like characteristics overlap for these model compounds (100% in 4 out of the 5 sets of rules, the same matches in the drugability rules exhibited by naturally degraded limonoids). Thus, in theory, they could be biologically relevant compounds. Furthermore, the physicochemical descriptors for compounds **1**–**3** seem to predict better ADMET characteristics in comparison to those expected for **swietenolide** and its derivative **2-hydroxy-3-*O*-tigloylswietenolide**, which may be remarkable for preparing new drugs.

These model molecules **1-3** were synthetized and tested against *S. aureus* ATCC 25923 and MRSA strains. Assays did not show the antibacterial activity of (±)-**3a** against *S. aureus* and MRSA strains. The free hydroxyl-containing stereoisomers (±)-**1a** and (±)-**1b** displayed low activity against all the tested bacteria (MIC_50_ = 512 mg/L) while acetate derivatives (±)-**2a** and (±)-**2b** were more active showing MIC_50_ = 16 mg/L against *S. aureus* and values below 100 mg/L against MRSA 15019301 and MRSA 18032913. Stereoisomer mixture **2** was the most active of them. Because of this reason, it was also tested against other Gram-positive bacteria (Table 4). Once again, this mixture exhibit antibacterial effect against linezolid-resistant *Staphylococcus epidermidis* clinical isolates (MIC_50_ = 64 mg/L) and lower activity against *Listeria monocytogenes* (MIC_50_ = 128 mg/L), *Enterococcus faecalis* ATCC 29212 (MIC_50_ = 256 mg/L), and *Enterococcus* spp. clinical isolates (daptomycine-resistant *E. faecalis* and vancomycin-resistant *E. faecium*, showing both MIC_50_ = 256 mg/L).

These results encouraged us to design derivatives that could be more active, and lipophilicity was considered as a key descriptor. Some authors suggest that lipophilicity is one of the most important molecular properties to be considered on decision-making in medicinal chemistry [19], and certain studies pointed out that high hydrophilicity decreases the biological activity of certain natural products and, as a consequence, higher lipophilicity, a higher activity [12]. Lipophilicity is represented by the descriptors partition coefficient (log P)—which is often used in the analysis of structure-activity relationships (SAR and QSAR) [49,50,51,52]—and distribution coefficient (log D) that is considered as the most impactful parameter by some authors, rather than log P [52]. 

In the preliminary antibacterial assays, stereroisomer mixture of **2** exhibited much lower MIC_50_ value (16 mg/L) than the hydroxylated compounds **1** (512 mg/L) against *S. aureus* ATCC 25923. The acetylation of the free hydroxyl group of **1** to produce **2** involves a higher lipophilicity for **2**. 

Considering the previous, we proposed to introduce longer side-chains in the ester group to assess if the antibacterial activity could be positively affected. Starting from the mixture (±)-**1a** and (±)-**1b**, a set of esters containing butanoate ((±)-**4a** and (±)-**4b**), hexanoate ((±)-**5a** and (±)-**5b**)), and decanoate chains ((±)-**6a** and (±)-**6b**)) (Scheme 3) were proposed to be synthetized for obtaining a gradual increase of lipophilicity (Table 6) at the same time they conform in the druggability rules previously mentioned (Table S2).

Curiously—contrary to our hypothesis—the higher lipophilic character of molecules 4–6 respect to the previous molecules assayed (1-2), decreased the antibacterial activity against *S. aureus* ATCC 25923. For compounds 4–6 evidenced that the less polar enantiomer pairs a ((±)-4a-(±)-6a) were notably less active than enantiomeric pairs b ((±)-4b-(±)-6b) (Table 5). Stereochemical factors could play a key role in the inhibition process. 

The results demonstrated that amongst the analyzed compounds 1–6, acetylated mixture 2 was the most active of them all against *S. aureus* ATCC 25923 and the acetate derivatives 2 could be further explored to develop new antimicrobial drugs to combat *S. aureus* bacterial infections.

## 4. Materials and Methods

### 4.1. General

Unless otherwise noted, materials and reagents were obtained from commercial suppliers and were used without further purification. Infrared spectra were recorded on an FT-IR spectrophotometer and reported as the wave number (cm^−1^). ^1^H and ^13^C NMR measurements were recorded on Agilent 500 MHz NMR spectrometer with SiMe_4_ as the internal reference. Chemical shifts were referenced to CDCl_3_ (*δ*_H_ 7.25, *δ*_C_ 77.0). NMR assignments were made using a combination of 1D and 2D techniques. High-resolution mass spectroscopy (HRMS) was performed in a QTOF mass spectrometer in the positive ion ESI mode. Purification by analytical HPLC was performed with a Hitachi/Merck L-6270 apparatus equipped with a differential refractometer detector (RI-7490). A LiChrospher^®^ Si gel 60 (10 µm) LiChroCart^®^ (250 mm × 4 mm) were used in isolation experiments. Silica gel (Merck) was used for column chromatography. TLC was performed on Merck Kiesegel 60 F254, 0.25 mm thick.

### 4.2. Reaction Procedures

#### 4.2.1. Preparation of 6-(furan-3-yl-(hydroxy)methyl)cyclohex-2-en-1-one (Mixture of Stereoisomers (±)-1a and (±)-1b)

A solution of *n*-BuLi (1.6 M in hexane, 11.4 mL, 18.2 mmol) was added dropwise at –50 °C to a solution of *N*,*N*-diisopropylamine (2.6 mL, 19.08 mmol) in dry THF (22 mL) under an argon atmosphere to prepare lithium diisopropylamide (LDA) in situ. Then cyclohex-2-en-1-one (1 g, 10.4 mmol) was added dropwise and, 3-furaldehyde (0.92 mL, 10.40 mmol) resulting in a pale yellow solution. The mixture was stirred for 3 h at –50 °C, and then was allowed to warm to room temperature. Then, water was added (10 mL), the layers were separated and the aqueous layer was extracted with ethyl acetate (3 × 50 mL). The combined organic layers were dried over anhydrous sodium sulfate. Filtration and evaporation of the solvent under reduced pressure yielded the crude material that was purified by silica gel chromatography to give (±)-1a and (±)-1b (1.37 g, 69% yield).

*6-(furan-3-yl-(hydroxy)methyl)cyclohex-2-en-1-one* (mixture of stereoisomers (±)-1a and (±)-1b). Amorphous solid. IR (KBr) *ν*_máx_ (cm^−1^): 3439 (ν O-H), 2929 (ν C-H), 1667 (ν C=O *(α*,*β*-unsaturated ketone)),1160, 1024, 874, 602; ^1^H NMR *δ* (CDCl_3_, 500 MHz): 7.41–7.36 (4H, m, H-2′ and H-5′), 7.05–6.97 (2H, m, H-3), 6.42 (1H, dd, *J* = 1.9, 0.9 Hz, H-4′), 6.29 (1H, t, *J* = 1.4 Hz, H-4′), 6.08–6.02 (2H, m, H-2), 5.28 (1H, dd, *J* = 6.4, 3.2 Hz, C*H*OH), 4.89 (1H, dd, *J* = 8.5, 1.9 Hz, C*H*OH), 4.55 (d, *J* = 1.9 Hz, O*H*), 3.20 (d, *J* = 6.4 Hz, O*H*), 2.70–2.62 (1H, m, H-6), 2.55 (1H, ddd, *J* = 13.5, 8.5, 4.7 Hz, H-6), 2.49–2.34 (4H, m, H-4), 1.93–1.85 (2H, m, H-5), 1.84–1.75 (1H, m, H-5a), 1.63–1.51 (1H, m, H-5b); ^13^C NMR *δ* (CDCl_3_, 125 MHz): 203.3 (s, C-1), 201.3 (s, C-1), 151.6 (d, C-3), 151.3 (d, C-3), 143.4 (d, C-5′), 143.1 (d, C-5′), 140.2 (d, C-2′), 139.7 (d, C-2′), 130.0 (d, C-2), 129.6 (d, C-2), 126.1 (s, C-3′), 125.6 (s, C-3′), 108.8 (d, C-4′), 108.6 (d, C-4′), 67.7 (d, *C*HOH), 66.5 (d, *C*HOH), 52.0 (d, C-6), 51.9 (d, C-6), 25.9 (t, C-4), 25.8 (t, C-4), 25.3 (t, C-5), 22.8 (t, C-5); HRMS (ESI+): calcd for C_11_H_13_O_3_ [M+H]^+^ 193.0865, found 193.0846. 

#### 4.2.2. Preparation of furan-3-yl-(2-oxocyclohex-3-en-1-yl)-methyl acetate (Mixture of Stereoisomers (±)-2a and (±)-2b)

Pyridine (2 drops) was added to a solution of the mixture of stereoisomers (±)-1a and (±)-1b (100 mg, 0.52 mmol) in acetic anhydride (0.5 mL) at room temperature for 18 h. Then, cyclohexane was added (2 mL) and the solvent was evaporated under reduced pressure. This procedure was repeated three times to give (±)-2a and (±)-2b in >99% yield.

*Furan-3-yl-(2-oxocyclohex-3-en-1-yl)-methyl acetate* (mixture of stereoisomers (±)-2a and (±)-2b). Yellow oil. IR (KBr) *ν*_máx_ (cm^−1^): 2938 (ν C-H), 1745 (ν C=O, aliphatic ketone), 1675 (ν C=O *(α*,*β*-unsaturated ketone)), 1239, 1025, 875, 602; ^1^H NMR *δ* (CDCl_3_, 500 MHz): 7.40–7.32 (4H, m, H-2′ and H-5′), 6.96 (1H, dddd, *J* = 9.6, 5.2, 2.9, 0.9 Hz, H-4″), 6.90 (1H, dddd, *J* = 10.0, 4.6, 3.3, 0.9 Hz, H-4″), 6.46 (1H, d, *J* = 4.2 Hz, C*H*OCO), 6.44 (1H, d, *J* = 5.8 Hz, C*H*OCO), 6.33 (1H, dd, *J* = 1.8, 0.8 Hz, H-4′), 6.31 (1H, brs, H-4′), 6.03 (1H, ddd, *J* = 9.6, 2.7, 1.4 Hz, H-3″), 5.97 (1H, dt, *J* = 10.0, 1.9 Hz, H-3″), 2.86 (1H, ddd, *J* = 11.6, 5.8, 4.6 Hz, H-1″), 2.63 (1H, dt, *J* = 11.7, 4.2 Hz, H-1″), 2.51–2.29 (4H, m, H-5″), 2.13–2.06 (2H, m, H-6″a), 2.05 (3H, s, H-2), 2.01 (3H, s, H-2), 1.76–1.64 (2H, m, H-6″b); ^13^C NMR *δ* (CDCl_3_, 125 MHz): 197.8 (s, C-2″), 197.1 (s, C-2″), 169.7 (s, C-1), 166.4 (s, C-1), 150.1 (d, C-4″), 149.7 (d, C-4″), 143.2 (d, C-5′), 142.9 (d, C-5′), 140.6 (d, C-2′), 139.8 (d, C-2′), 129.8 (d, C-3″), 129.6 (d, C-3″), 123.7 (s, C-3′), 122.2 (s, C-3′), 109.3 (d, C-4′), 108.9 (d, C-4′), 67.5 (d, *C*HOCO), 66.4 (d, *C*HOCO), 51.0 (d, C-1″), 50.6 (d, C-1″), 25.3 (t, C-5″), 24.9 (t, C-5″), 23.5 (t, C-6″), 22.7 (t, C-6″), 21.1 (q, C-2), 20.9 (q, C-2); HRMS (ESI+): calcd for C_13_H_14_O_4_Na [M+Na]^+^ 257.0790, found 257.0790.

#### 4.2.3. Preparation of (1S*,8aS*)-1-(furan-3-yl)-4a-hydroxy-1,4,4a,7,8,8a-hexahydro-3H-isocromen-3-one ((±)-3a)

A solution of the stereoisomer mixture (±)-2a and (±)-2b (50 mg, 0.22 mmol) in dry THF (0.6 mL) was added dropwise to the LDA solution (0.26 mmol) at –50 °C. The reaction mixture was stirred at –50 °C for 4 h, and then saturated aqueous NH_4_CI (5 mL) was added and the mixture was stirred and gradually warmed to room temperature. The layers were separated and the aqueous layer was extracted with ethyl acetate (3 × 20 mL). The combined organic layers were dried over anhydrous sodium sulfate. Filtration and evaporation of the solvent under reduced pressure yielded the crude material that was purified by silica gel chromatography to give (±)-3a (3.5 mg, 7% yield).

(1S*,8aS*)-1-(furan-3-yl)-4a-hydroxy-1,4,4a,7,8,8a-hexahydro-3H-isochromen-3-one ((±)-3a). Yellow oil. IR (KBr) *ν*_máx_ (cm^−1^): 3419 (ν O-H), 2933 (ν C-H), 1732 (ν C=O, aliphatic ketone), 1022, 875, 755, 603; ^1^H NMR *δ* (CDCl_3_, 500 MHz): 7.50 (brs, H-2′, 1H), 7.45 (1H, d, *J* = 1.8 Hz, H-5′), 6.50 (1H, dd, *J* = 1.8, 0.9 Hz, H-4′), 5.99 (1H, dt, *J* = 9.9, 3.9 Hz, H-6), 5.68 (1H, dt, *J* = 9.9, 2.1 Hz, H-5), 4.94 (1H, d, *J* = 10.0 Hz, H-1), 2.77 (1H, d, *J* = 16.0 Hz, H-4*α*),^†^ 2.72 (1H, d, *J* = 16.0 Hz, H-4*β*),^†^ 2.26 (1H, ddd, *J* = 10,0, 7.9, 4.4 Hz, H-8a), 2.07–2.00 (2H, m, H-7), 1.87 (1H, ddt, *J* = 13.5, 6.3, 4.4 Hz, H-8*α*),^‡^ 1.44 (1H, ddt, *J* = 13.5, 7.9, 5.9 Hz, H-8*β*)^‡^.^†,‡^Interchangeable signals; ^13^C NMR *δ* (CDCl_3_, 125 MHz): 170.2 (s, C-3), 144.0 (d, C-5′), 140.8 (d, C-2′), 131.4 (d, C-6), 130.3 (d, C-5), 123.1 (s, C-3′), 108.7 (d, C-4′), 75.2 (d, C-1), 68.6 (s, C-4a), 45.9 (d, C-8a), 42.9 (t, C-4), 22.6 (t, C-8), 22.1 (t, C-7); HRMS (ESI+): calcd for C_13_H_14_O_4_Na [M+Na]^+^ 257.0790, found 257.0794.

#### 4.2.4. General Procedure for Steglich Esterification. Preparation of (±)-4a, (±)-4b, (±)-5a and (±)-5b

A mixture of 6-(furan-3-yl-(hydroxy)methyl)cyclohex-2-en-1-one (mixture of stereoisomers (±)-1a and (±)-1b) (100 mg, 0.43 mmol), the corresponding carboxylic acid (butanoic acid (0.06 mL; 0.64 mmol) or hexanoic acid (0.08 mL; 0.64 mmol)) and dimethylaminopyridine (DMAP) (10.50 mg, 0.09 mmol) in dry CH_2_Cl_2_ (6.5 mL) was stirred for 10 min. To the resulting mixture was added *N*-(3-dimethylaminopropyl)-*N*’-ethylcarbodiimide (EDC) (98 mg, 0.52 mmol). After stirring for 18 h, the reaction mixture was evaporated and then added Et_2_O (10 mL). The crude was sequentially washed with H_2_O (10 mL), twice with saturated sodium bicarbonate solution (10 mL), H_2_O (10 mL), dried over anhydrous sodium sulphate, filtered and evaporated under reduced pressure to afford the stereoisomer mixture (±)-4a-(±)-4b, and (±)-5a-(±)-5b. The crude material of 4 was purified by HPLC to give (±)-4a and (±)-4b as single diastereoisomers (27.3 mg, 20% yield). The crude material of 5 was purified by silica gel chromatography to yield (±)-5a and (±)-5b as single diastereoisomers (84.5 mg, 56% yield).

*Furan-3-yl-(2-oxocyclohex-3-en-1-yl)methyl butyrate* (diastereoisomer (±)-4a). (13.4 mg, 10.2% yield of isolated product). Colorless oil. *t*_R_ = 12 min, petroleum ether: ethyl acetate (80: 20), flow = 1.0 mL.min^−1^; R_f_: 0.55 (petroleum ether: AcOEt (3: 1)); IR (KBr) *ν*_máx_ (cm^−1^): 2964 (ν C-H), 2933 (ν C-H), 1738 (ν C=O, aliphatic ketone), 1677 (ν C=O *(α*,*β*-unsaturated ketone)), 1168, 1025, 875, 601; ^1^H NMR *δ* (CDCl_3_, 500 MHz): 7.38 (1H, dt, *J* = 1.6, 0.8 Hz, H-2′), 7.34 (1H, t, *J* = 1.6 Hz, H-5′), 6.94–6.88 (1H, m, H-4″), 6.46 (1H, d, *J* = 5.7 Hz, C*H*OCO), 6.34 (1H, brs, H-4′), 5.98 (1H, ddd, *J* = 10.1, 2.4, 1.6 Hz, H-3″), 2.87 (1H, ddd, *J* = 11.6, 5.7, 4.6 Hz, H-1″), 2.40–2.34 (2H, m, H-5″), 2.29 (2H, t, *J* = 7.4 Hz, H-2), 2.13–2.05 (2H, m, H-6″), 1.65 (2H, sext, *J* = 7.4 Hz, H-3), 0.93 (3H, t, *J* = 7.4 Hz, H-4); ^13^C NMR *δ* (CDCl_3_, 125 MHz): 197.8 (s, C-2″), 172.4 (s, C-1), 149.6 (d, C-4″), 142.9 (d, C-5′), 140.6 (d, C-2′), 129.7 (d, C-3″), 122.3 (s, C-3′), 109.4 (d, C-4′), 67.3 (d, *C*HOCO), 50.7 (d, C-1″), 36.4 (t, C-2), 24.9 (t, C-5″), 23.5 (t, C-6″), 18.4 (t, C-3), 13.6 (q, C-4); M.S. (E.I.) *m/z* (relative intensity): 191.1 (28.0), 174.1 (28.5), 123.1 (32.3), 96.1 (64.8), 95.0 (100), 77.7 (45.7), 71.1 (48.3),68.1 (30.1), 67.2 (42.2), 43.2 (98.7), 41.2 (64.3); HRMS (ESI+): calcd for C_15_H_18_O_4_Na [M+Na]^+^ 285.1103, found 285.1101.

*Furan-3-yl-(2-oxocyclohex-3-en-1-yl)methyl butyrate* (diastereoisomer (±)-4b). (13.9 mg, 9.8% yield of isolated product). Colorless oil. *t*_R_ = 14 min, petroleum ether: ethyl acetate (80: 20), flow = 1.0 mL.min^−1^; R_f_: 0.48 (petroleum ether: AcOEt (3: 1)); IR (KBr) *ν*_máx_ (cm^−1^): 2964 (ν C-H), 2933 (ν C-H), 1738 (ν C=O, aliphatic ketone), 1677 (ν C=O *(α*,*β*-unsaturated ketone)), 1168, 1025, 875, 601; ^1^H NMR *δ* (CDCl_3_, 500 MHz): 7.36 (1H, t, *J* = 1.8 Hz, H-5′), 7.33 (1H, dt, *J* = 1.8, 0.9 Hz, H-2′), 7.00–6.94 (1H, m, H-4″), 6.48 (1H, d, *J* = 4.2 Hz, C*H*OCO), 6.32 (1H, dd, *J* = 1.8, 0.9 Hz, H-4′), 6.03 (1H, ddd, *J* = 10.0, 2.6, 1.4 Hz, H-3″), 2.64 (1H, dt, *J* = 11.6, 4.2 Hz, H-1″), 2.52–2.43 (1H, m, H-5″a), 2.41–2.30 (1H, m, H-5″b), 2.26 (2H, t, *J* = 7.4 Hz, H-2), 2.15–2.05 (2H, m, H-6″), 1.62 (2H, sext, *J* = 7.4 Hz, H-3), 0.86 (3H, t, *J* = 7.4 Hz, H-4); ^13^C NMR *δ* (CDCl_3_, 125 MHz): 197.0 (s, C-2″), 172.2 (s, C-1), 150.0 (d, C-4″), 143.2 (d, C-5′), 139.8 (d, C-2′), 129.9 (d, C-3″), 123.9 (s, C-3′), 109.0 (d, C-4′), 66.2 (d, *C*HOCO), 51.1 (d, C-1″), 36.2 (t, C-2), 25.3 (t, C-5″), 22.8 (t, C-6″), 18.5 (t, C-3), 13.6 (q, C-4); M.S. (E.I.) *m/z* (relative intensity): 191.1 (28.5), 174.1 (30.9), 96.1 (58.8), 95.0 (86.5), 77.1 (29.3), 71.1 (47.0), 65.4 (33.6), 43.2 (98.8), 41.3 (100); HRMS (ESI+): calcd for C_15_H_18_O_4_Na [M+Na]^+^ 285.1103, found 285.1101.

*Furan-3-yl-(2-oxocyclohex-3-en-1-yl)methyl hexanoate* (diastereoisomer (±)-5a). (43.2 mg, 28.6% yield of isolated product). Colorless oil. R_f_: 0.67 (petroleum ether: AcOEt (3: 1)); IR (KBr) *ν*_máx_ (cm^−1^): 2956 (ν C-H), 2932 (ν C-H), 1738 (ν C=O, aliphatic ketone), 1676 (ν C=O *(α*,*β*-unsaturated ketone)), 1163, 1023, 874, 602; ^1^H NMR *δ* (CDCl_3_, 500 MHz): 7.38 (1H, dt, *J* = 1.6, 0.8 Hz, H-5′), 7.34 (1H, m, H-2′), 6.91 (1H, dddd, *J* = 10.0, 4.6, 3.2, 1.0 Hz, H-4″), 6.46 (1H, d, *J* = 5.7 Hz, C*H*OCO), 6.36–6.31 (1H, m, H-4′), 5.97 (1H, ddd, *J* = 10.0, 2.3, 1.4 Hz, H-3″), 2.87 (1H, ddd, *J* = 11.6, 5.7, 4.5 Hz, H-1″), 2.40–2.34 (2H, m, H-5″), 2.33–2.29 (2H, m, H-2), 2.13–2.05 (2H, m, H-6″), 1.65–1.56 (2H, m, H-3), 1.32–1.26 (4H, m, H-4 and H-5), 0.91 (3H, t, *J* = 7.4 Hz, H-6). ^13^C NMR *δ* (CDCl_3_, 125 MHz): 197.8 (s, C-2″), 172.6 (s, C-1), 149.6 (d, C-4″), 142.9 (d, C-5′), 140.6 (d, C-2′), 129.7 (d, C-3″), 122.3 (s, C-3′), 109,4 (d, C-4′), 67.3 (d, *C*HOCO), 50.7 (d, C-1″), 34.4 (t, C-2), 31.2 (t, C-4),^¥^, 24.9 (t, C-5″), 24.6 (t, C-3), 23.5 (t, C-6″), 22.3 (t, C-5),^¥^, 13.9 (q, C-6) ^¥^Interchangeable signals; M.S. (E.I.) *m/z* (relative intensity): 191.1 (54.0), 174.1 (56.0), 123.1 (35.5), 96.1 (71.8), 95.0 (100), 71.2 (43.3), 43.2 (63.4), 41.2 (27.3); HRMS (ESI+): calcd for C_17_H_22_O_4_Na [M+Na]^+^ 313.1416, found 313.1417.

*Furan-3-yl-(2-oxocyclohex-3-en-1-yl)methyl hexanoate* (diastereoisomer (±)-5b). (41.3 mg, 27.4% yield of isolated product). Colorless oil. R_f_: 0.58 (petroleum ether: AcOEt (3: 1)); IR (KBr) *ν*_máx_ (cm^−1^): 2956 (ν C-H), 2932 (ν C-H), 1738 (ν C=O, aliphatic ketone), 1676 (ν C=O *(α*,*β*-unsaturated ketone)), 1163, 1023, 874, 602; ^1^H NMR *δ* (CDCl_3_, 500 MHz): 7.36 (1H, d, *J* = 1.8 Hz, H-5′), 7.33 (1H, d, *J* = 0.8 Hz, H-2′), 7.00–6.94 (1H, m, H-4″), 6.48 (1H, d, *J* = 4.3 Hz, C*H*OCO), 6.32 (1H, dd, *J* = 1.8, 0.8 Hz, H-4′), 6.05–6.01 (1H, m, H-3″), 2.64 (1H, dt, *J* = 11.6, 4.3 Hz, H-1″), 2.53–2.43 (1H, m, H-5″), 2.40–2.30 (1H, m, H-5″), 2.27 (2H, t, *J* = 7.4 Hz, H-2), 2.15–2.00 (2H, m, H-6″), 1.60 (2H, quint, *J* = 7.4 Hz, H-3), 1.32–1.20 (4H, m, H-4 and H-5), 0.87 (3H, t, *J* = 7.4 Hz, H-6). ^13^C NMR *δ* (CDCl_3_, 125 MHz): 197.0 (s, C-2″), 172.4 (s, C-1), 150.0 (d, C-4″), 143.2 (d, C-5′), 139.8 (d, C-2′), 129.9 (d, C-3″), 123.9 (s, C-3′), 109.0 (d, C-4′), 66.2 (d, *C*HOCO), 51.1 (d, C-1″), 34.3 (t, C-2), 31.2 (t, C-4),^¥^ 25.3 (t, C-5″), 24.6 (t, C-3), 22.8 (t, C-6″), 22.3 (t, C-5),^¥^ 13.9 (q, C-6) ^¥^Interchangeable signals; M.S. (E.I.) *m/z* (relative intensity): 191.1 (43.9), 174.1 (49.5), 123.1 (32.9), 96.1 (58.1), 95.0 (100), 71.2 (37.1), 43.2 (56.3), 41.2 (26.0); HRMS (ESI+): calcd for C_17_H_22_O_4_Na [M+Na]^+^ 313.1416, found 313.1417.

#### 4.2.5. Preparation of (±)-6a and (±)-6b

Triethylamine (0.18 mL; 1.29 mmol) was added to a solution of a mixture of 6-(furan-3-yl-(hydroxy)methyl)cyclohex-2-en-1-one (mixture of stereoisomers (±)-1a and (±)-1b) (100 mg, 0.43 mmol) in dry CH_2_Cl_2_ (5 mL). The reaction mixture was stirred at room temperature for 15 min. To the resulting mixture was added decanoyl chloride (0.14 mL, 0.64 mmol). After stirring for 18 h, H_2_O (5 mL) was added to the reaction mixture. The crude was sequentially washed with H_2_O (10 mL), twice with brine (10 mL), H_2_O (10 mL), dried over anhydrous sodium sulphate, filtered and evaporated under reduced pressure to afford the stereoisomer mixture (±)-6a and (±)-6b. The crude material was purified by silica gel chromatography to yield (±)-6a and (±)-6b as single diastereoisomers (79.3 mg, 44% yield).

*Furan-3-yl-(2-oxocyclohex-3-en-1-yl)methyl decanoate* (diastereoisomer (±)-6a). (41.3 mg, 22.9% yield of isolated product). Colorless oil. R_f_: 0.44 (petroleum ether: AcOEt (9: 1)); IR (KBr) *ν*_máx_ (cm^−1^): 2953 (ν C-H), 2925 (ν C-H), 1743 (ν C=O, aliphatic ketone), 1681 (ν C=O *(α*,*β*-unsaturated ketone)), 1161, 1026, 875, 723, 602; ^1^H NMR *δ* (CDCl_3_, 500 MHz): 7.38 (1H, dt, *J* = 1.7, 0.8 Hz, H-2′), 7.33 (1H, t, *J* = 1.7 Hz, H-5′), 6.91 (1H, dddd, *J* = 10.1, 4.6, 3.3, 1.0 Hz, H-4″), 6.46 (1H, d, *J* = 5.7 Hz, C*H*OCO), 6.33 (1H, dd, *J* = 1.7, 0.8 Hz, H-4′), 5.98 (1H, ddd, *J* = 10.1, 2.4, 1.6 Hz, H-3″), 2.87 (1H, ddd, *J* = 11.7, 5.7, 4.5 Hz, H-1″), 2.41–2.33 (1H, m, H-5″a), 2.33–2.29 (2H, m, H-2), 2.15–2.04 (1H, m, H-6″a), 1.76–1.66 (3H, m, H-3 and H-6″b), 1.65–1.56 (1H, m, H-5″b), 1.31–1.21 (12H, m, H-4, H-5, H-6, H-7, H-8 and H-9), 0.87 (3H, t, *J* = 6.8 Hz, H-10); ^13^C NMR *δ* (CDCl_3_, 125 MHz): 197.7 (s, C-2″), 172.6 (s, C-1), 149.6 (d, C-4″), 142.9 (d, C-5′), 140.6 (d, C-2′), 129.7 (d, C-3″), 122.3 (s, C-3′), 109.4 (d, C-4′), 67.3 (d, *C*HOCO), 50.7 (d, C-1″), 34.5 (t, C-2), 31.8 (t, C-4),^¥^ 29.4 (t, C-5),^¥^ 29.2 (t, C-6 and C-7, 2C),^¥^ 29.1 (t, C-8),^¥^ 24.9 (t, C-3 and C-5″, 2C), 23.5 (t, C-6″), 22.7 (t, C-9),^¥^ 14.1 (q, C-10).^¥^Interchangeable signals; M.S. (E.I.) *m/z* (relative intensity): 191.1 (52.2), 174.1 (44.6), 123.1 (43.5), 96.1 (64.6), 95.1 (100), 57.2 (36.9), 55.1 (43.6), 43.2 (65.7), 41.2 (65.2); HRMS (ESI+): calcd for C_21_H_30_O_4_Na [M+Na]^+^ 369.2042, found 369.2053.

*Furan-3-yl-(2-oxocyclohex-3-en-1-yl)methyl decanoate* (diastereoisomer (±)-6b). (38.0 mg, 21.1% yield of isolated product). Colorless oil. R_f_: 0.36 (petroleum ether: AcOEt (9: 1)); IR (KBr) *ν*_máx_ (cm^−1^): 2953 (ν C-H), 2925 (ν C-H), 1743 (ν C=O, aliphatic ketone), 1681 (ν C=O *(α*,*β*-unsaturated ketone)), 1161, 1026, 875, 723, 602; ^1^H NMR *δ* (CDCl_3_, 500 MHz): 7.36 (1H, t, *J* = 1.6 Hz, H-5′), 7.33 (1H, dd, *J* = 1.6, 0.9 Hz, H-2′), 6.97 (1H, ddd, *J* = 10.0, 5.0, 2.7 Hz, H-4″), 6.47 (1H, d, *J* = 4.2 Hz, C*H*OCO), 6.32 (1H, brs, H-4′), 6.03 (1H, ddd, *J* = 10.0, 2.7, 1.4 Hz, H-3″), 2.64 (1H, dt, *J* = 11.5, 4.2 Hz, H-1″), 2.52–2.43 (1H, m, H-5″a), 2.40–2.30 (1H, m, H-5″b), 2.27 (2H, t, *J* = 7.8 Hz, H-2), 2.14–2.04 (2H, m, H-6″), 1.62–1.55 (2H, m, H-3), 1.31–1.21 (12H, m, H-4, H-5, H-6, H-7, H-8 and H-9), 0.87 (3H, t, *J* = 6.8 Hz, H-10). ^13^C NMR *δ* (CDCl_3_, 125 MHz): 196.8 (s, C-2″), 172.6 (s, C-1), 150.3 (d, C-4″), 143.3 (d, C-5′), 140.0 (d, C-2′), 129.8 (d, C-3″), 123.1 (s, C-3′), 108.8 (d, C-4′), 67.1 (d, *C*HOCO), 51.0 (d, C-1″), 34.8 (t, C-2), 31.9 (t, C-4),^¥^ 29.6 (t, C-5),^¥^ 29.2 (t, C-6 and C-7, 2C),^¥^ 29.1 (t, C-8),^¥^ 24.9 (t, C-3 and C-5″, 2C), 23.4 (t, C-6″), 22.6 (t, C-9),^¥^ 14.1 (q, C-10). ^¥^Interchangeable signals; M.S. (E.I.) *m/z* (relative intensity): 191.1 (46.1), 174.1 (44.6), 123.1 (40.4), 96.1 (53.7), 95.1 (100), 57.2 (36.9), 55.2 (43.8), 43.2 (64.1), 41.2 (64.4); HRMS (ESI+): calcd for C_21_H_30_O_4_Na [M+Na]^+^ 369.2042, found 369.2053.

### 4.3. Bacterial Strains

For the antimicrobial evaluation, two strains from the American Type Culture Collection (*S. aureus* ATCC 25923 and *E. faecalis* ATCC 29212), as well as, 9 clinical isolates (MRSA 15012406, 15019301, 17045463 and 18032913, daptomycine-resistant *E. faecalis* 16028257, vancomycin-resistant *E. faecium* 16051635, linezolid-resistant *S. epidermidis* 17101107 and 18031123, and *L. monocytogenes* 17035151) were used in this study.

### 4.4. In Vitro Susceptibility Testing

The minimal inhibitory concentration (MIC) was determined on 96 well culture plates by a microdilution method according to Clinical and Laboratory Standards Institute (CLSI) procedures [53], using Mueller–Hinton Broth (Sigma, Spain). 50 µL of inoculum (5 × 10^5^ CFU/mL) was added to each well. Proper blanks were assayed simultaneously. The MIC was determined as the lowest concentration, which showed no visible growth. Fosfomycin was used as a reference standard. The procedures were performed in independent triplicates to validate the results.

### 4.5. Time–Kill Kinetic Assays

Time–kill curves of *S. aureus* ATCC 25923 and MRSA isolate 18032913 were carried out following the procedure described by CLSI [53]. Concentration equal to MIC, twice the MIC and four times the MIC of the extracts were prepared. An inoculum size of 2 × 10^6^ CFU/mL was conducted on Mueller Hinton broth (Sigma, Spain). Aliquots of 0.5 mL of the medium were taken at time intervals of 0, 4, 8 10, 12, and 24h. A graph of the log CFU/mL was plotted against time.

## Figures and Tables

**Table 1:** Calculated physicochemical properties of the naturally-occurring limonoids **swietenolide** and **2-hydroxy-3-*O*-tigloylswietenolide** by ADMETlab [28].

Molecular Properties	Limonoids		Molecular Properties	Limonoids	
	Swietenolide	2-hydroxy-3-*O*-tigloylswietenolide		Swietenolide	2-hydroxy-3-*O*-tigloylswietenolide
HBD ^a^	2	2	Nrotb ^g^	4	7
HBA ^b^	8	10	TPSA ^h^	123.27	149.57
MW ^c^	486.56	584.66	Log D ^i^	1.679	1.674
Log P ^d^	3.127	3.759	NR ^j^	5	5
MR ^e^	122.54	147.30	NRigB ^k^	35	39
TNA ^f^	69	82			
**Matches in Drugability Rules**					
**Rules**	**Lipinski**	**Ghose**	**Veber**	**Varma**	**Oprea**
Swietenolide	100%	75%	100%	80%	66.7%
2-hydroxy-3-*O*-tigloylswietenolide	75%	25%	66.7%	40%	100%

^a^ HBD = hydrogen bond donors; ^b^ HBA = hydrogen bond acceptors; ^c^ MW = molecular weight; ^d^ Log P = partition coefficient; ^e^ MR = molar refractivity; ^f^ TNA = total number of atoms; ^g^ Nrotb = number of rotatable bonds; ^h^ TPSA = total polar surface area; ^i^ Log D = distribution coefficient; ^j^ NR = number of rings; k) NRigB = number of rigid bonds.

**Table 2:** Calculated physicochemical properties of the naturally-occurring degraded limonoids **dictamdiol, calodendrolide, fraxinellone, 8,14-epoxyfraxinellone,** and **melazolide A** by ADMETlab [28].

Molecular Properties	Natural Degraded Limonoids				
	Dictamdiol	Calodendrolide	Fraxinellone	8,14-epoxyfraxinellone	Melazolide A
HBD ^a^	2	0	0	0	2
HBA ^b^	5	4	4	4	4
MW ^c^	278.304	260.289	246.262	248.278	212.245
Log P ^d^	1.716	2.762	2.563	2.595	0.238
MR ^e^	69.596	66.243	62.589	61.72	52.944
TNA ^f^	38	35	32	34	31
Nrotb ^g^	1	1	1	1	1
TPSA ^h^	79.9	51.97	56.51	51.97	66.76
Log D ^i^	0.978	1.513	1.383	1.374	0.189
NR ^j^	3	4	3	4	2
NRigB ^k^	21	21	19	20	15
**Rules**	**Matches**				
Lipinski’s rules	100%	100%	100%	100%	100%
Ghose’s rules	100%	100%	100%	100%	100%
Veber’s rules	100%	100%	100%	100%	100%
Varma’s rules	100%	100%	100%	100%	100%
Oprea’s rules	66.7%	66.7%	66.7%	66.7%	0%

^a^ HBD = hydrogen bond donors; ^b^ HBA = hydrogen bond acceptors; ^c^ MW = molecular weight; ^d^ Log P = partition coefficient; ^e^ MR = molar refractivity; ^f^ TNA = total number of atoms; ^g^ Nrotb = number of rotatable bonds; ^h^ TPSA = total polar surface area; ^i^ Log D = distribution coefficient; ^j^ NR = number of rings; ^k^ NRigB = number of rigid bonds.

**Table 3:** Minimal inhibitory concentrations are effective for ≥50% for **1**–**3** against *S. aureus* ATCC 25923 and MRSA strains.

Pathogen	Stereoisomers	MIC_50_ (mg/L)
***S. aureus* ATCC 25923**	(±)-**1a** and (±)-**1b**	512
	(±)-**2a** and (±)-**2b**	16
	(±)-**3a**	ND ^1^
**MRSA 15012406**	(±)-**1a** and (±)-**1b**	512
	(±)-**2a** and (±)-**2b**	128
	(±)-**3a**	ND ^1^
**MRSA 15019301**	(±)-**1a** and (±)-**1b**	512
	(±)-**2a** and (±)-**2b**	64
	(±)-**3a**	ND ^1^
**MRSA 17045463**	(±)-**1a** and (±)-**1b**	NT ^2^
	(±)-**2a** and (±)-**2b**	128
	(±)-**3a**	NT ^2^
**MRSA 18032913**	(±)-**1a** and (±)-**1b**	NT ^2^
	(±)-**2a** and (±)-**2b**	64
	(±)-**3a**	NT ^2^

^1^ ND = not detectable; ^2^ NT = not tested.

**Table 4:** Minimal inhibitory concentrations are effective for ≥50% for **2** against other Gram-positive bacteria.

Pathogen	*L. monocytogenes* 17035151	*E. faecalis* ATCC 29212	*E. faecalis* DapR ^1^ 16028257	*E. faecium* VaR ^2^ 16051635	*S. epidermidis* LzdR ^3^ 17101107	*S. epidermidis* LzdR ^3^ 18031123
**MIC_50_** **(mg/L)**	128	256	256	256	64	64

^1^ DapR = daptomycine resistant; ^2^ VaR = vancomycin resistant; ^3^ LzdR = linezolid resistant.

**Table 5:** Minimal inhibitory concentrations are effective for ≥50% for 4–6 against *S. aureus* ATCC 25923.

Pathogen	Stereoisomers	MIC_50_ (mg/L)
*S. aureus* ATCC 25923	(±)-**4a**	256
	(±)-**4b**	128
	(±)-**5a**	256
	(±)-**5b**	128
	(±)-**6a**	1024
	(±)-**6b**	256

**Table 6:** Calculated log P and log D of 1 and esters 2 and 4-6 by ADMETlab [28].

Molecular Properties	Model Compounds				
	1	2	4	5	6
Log P	1.848	2.419	3.199	3.980	5.540
Log D	0.863	1.042	1.421	1.641	2.151

## Supplementary Materials

The following are available online at https://www.mdpi.com/2079-6382/9/8/488/s1, Table S1. Calculated physicochemical properties of the naturally-occurring limonoids swietenolide and 2-hydroxy-3-*O*-tigloylswietenolide by ADMETlab, Table S2. Calculated physicochemical properties of naturally degraded limonoids and the degraded limonoid analogs 1–6 by ADMETlab, Figure S1. NMR and HRMS Spectra of diastereoisomers (±)-1a and (±)-1b, Figure S2. NMR and HRMS Spectra of diastereoisomers (±)-2a and (±)-2b, Figure S3. NMR and HRMS Spectra of (±)-3a, Figure S4. NMR Spectra of diastereoisomers (±)-4a and (±)-4b, Figure S5. NMR Spectra of diastereoisomers (±)-5a and (±)-5b, Figure S6. NMR Spectra of diastereoisomers (±)-6a and (±)-6b, Figure S7. HRMS Spectra of compounds 4, 5, and 6.
