# Supplementary material for: Synthesis of Degraded Limonoid Analogs as New Antibacterial Scaffolds against Staphylococcus aureus
Source: Antibiotics (Basel). 2020 Aug 6;9(8):488. doi: 10.3390/antibiotics9080488 (PMC7459938; doi:10.3390/antibiotics9080488)
Supplement: Supplementary file 1 [file antibiotics-09-00488-s001.pdf]

SUPPLEMENTARY MATERIALS

Synthesis of Degraded Limonoid Analogs as New Antibacterial Scaffolds against *Staphylococcus aureus*

Marta Ferrera-Suanzes[1,‡], Victoria Prieto [2,‡], Antonio J. Medina-Olivera[1], José Manuel Botubol-Ares[1], Fátima Galán-Sánchez[2,3], Manuel A. Rodríguez Iglesias[2,3], Rosario Hernández-Galán[1,3] and María Jesús Durán-Peña [1,*]

[1] Department of Organic Chemistry, Faculty of Sciences, Campus Universitario Río San Pedro s/n, Torre Sur, 4ª planta, University of Cádiz, 11510, Puerto Real, Cádiz, Spain; marta.ferrerasuanzes@alum.uca.es (M. F. S.); antoniojesus.medinaolivera@alum.uca.es (A. J. M. O); josemanuel.botubol@uca.es (J. M. B. A.); rosario.hernandez@uca.es (R. H. G.)

[2] Department of Biomedicine, Biotechnology and Public Health, Hospital Puerta del Mar, University of Cádiz, 11009, Cádiz, Spain; vivprieto@gmail.com (V. P.); fatima.galan@uca.es (F. G. S.); manuel.rodriguezziglesias@uca.es (M. A. R. I.)

[3] Instituto de Investigación e Innovación Biomédica de Cádiz (INIBICA), Cádiz, 11009, Spain. fatima.galan@uca.es (F. G. S.); manuel.rodriguezziglesias@uca.es (M. A. R. I.); rosario.hernandez@uca.es (R. H. G.)

* Correspondence: mariajesus.duran@uca.es; Tel.: +34-956-016-583

‡ These authors contributed equally to this work.

Table of contents:

Table S1. Calculated physico-chemical properties of the naturally-occurring limonoids swietenolide and 2-hydroxy-3-*O*-tigloylswietenolide by ADMETlab ……………………….S3

Table S2. Calculated physico-chemical properties of natural degraded limonoids and the degraded limonoid analogs 1-6 by ADMETlab ……………………….……….………..…...S4

Figures S1. NMR and HRMS Spectra of diastereoisomers (±)-1a and (±)-1b……………..…….S6

Figure S1.1. ^{1}H NMR spectrum of diastereoisomers (±)-**1a** and (±)-**1b** in $CDCl_3$ (500 MHz)…. S6

Figure S1.2. ^{13}C NMR spectrum of diastereoisomers (±)-**1a** and (±)-**1b** in $CDCl_3$ (125 MHz)… S6

Figure S1.3. g-COSY spectrum of diastereoisomers (±)-**1a** and (±)-**1b** in $CDCl_3$………………...S7

Figure S1.4. HSQC spectrum of diastereoisomers (±)-**1a** and (±)-**1b** in $CDCl_3$…………………..S7

Figure S1.5. HMBC spectrum of diastereoisomers (±)-**1a** and (±)-**1b** in $CDCl_3$………………….S8

Figure S1.6. HRMS of **1** S8

Figures S2. NMR and HRMS Spectra of diastereoisomers (±)-2a and (±)-2b…………………....S9

Figure S2.1. ^{1}H NMR spectrum of diastereoisomers (±)-**2a** and (±)-**2b** in $CDCl_3$ (500 MHz) S9

Figure S2.2. ^{13}C NMR spectrum of diastereoisomers (±)-**2a** and (±)-**2b** in $CDCl_3$ (125 MHz) ... S9

Figure S2.3. HRMS of **2** S10

Figures S3. NMR and HRMS Spectra of (±)-3a…………………………..……….………..S10

Figure S3.1. ^{1}H NMR spectrum of (±)-**3a** in $CDCl_3$ (500 MHz) ………..……..…………….. S10

Figure S3.2. ^{13}C NMR spectrum of (±)-**3a** in $CDCl_3$ (125 MHz) S11

Figure S3.3. HRMS of (±)-**3a** S11

Figures S4. NMR Spectra of diastereoisomers (±)-4a and (±)-4b…………….........................S12

Figure S4.1. ^{1}H NMR spectrum of (±)-**4a** in $CDCl_3$ (500 MHz) ……………………………..... S12

Figure S4.2. ^{1}H NMR spectrum of (±)-**4b** in $CDCl_3$ (500 MHz) ……………………………..... S12

Figures S5. NMR Spectra of diastereoisomers (±)-5a and (±)-5b…………….........................S13

Figure S5.1. ^{1}H NMR spectrum of (±)-**5a** in $CDCl_3$ (500 MHz) ……………………………..... S13

Figure S5.2. ^{1}H NMR spectrum of (±)-**5b** in $CDCl_3$ (500 MHz) ……………………………..... S13

Figures S6. NMR Spectra of diastereoisomers (±)-6a and (±)-6b…………….........................S14

Figure S6.1. ^{1}H NMR spectrum of (±)-**6a** in $CDCl_3$ (500 MHz) ……………………………..... S14

Figure S6.2. ^{1}H NMR spectrum of (±)-**6b** in $CDCl_3$ (500 MHz) ……………………………..... S14

Figures S7. HRMS Spectra of compounds 4, 5 and 6 ……………………………………..…...S15

Figure S7.1. HRMS of 4 ……………………………………………..………………………….……S15

Figure S7.2. HRMS of 5 ……………………………...…………………………...……………….….S15

Figure S7.3. HRMS of 6 ………………….………………………………………….…………………S15

Table S1. Calculated physico-chemical properties of the naturally-occurring limonoids swietenolide and 2-hydroxy-3-*O*-tigloylswietenolide by ADMETlab.

**Molecular properties Lipinski's rules**	**Limonoids**	
	**swietenolide**	**2-hydroxy-3-*O*-tigloylswietenolide**
HBD[a] ≤ 5	2	2
HBA[b] ≤ 10	8	10
MW[c] ≤ 500 g/mol	486.56	584.66
Log P[d] ≤ 5	3.127	3.759
**Matches**	**100%**	**75%**
**Molecular properties Ghose's rules**	**Limonoids**	
	**swietenolide**	**2-hydroxy-3-*O*-tigloylswietenolide**
MW[c] ≤ 500 g/mol	486.56	584.66
Log P[d] ≤ 5	3.127	3.759
MR[e] 40<x <130	122.54	147.30
TNA[f] 20≤x≤70	69	82
**Matches**	**75%**	**25%**
**Molecular properties Veber's rules**	**Limonoids**	
	**swietenolide**	**2-hydroxy-3-*O*-tigloylswietenolide**
HBD[a] + HBA[b] ≤ 12	10	12
Nrotb[g] ≤ 10	4	7
TPSA[h] ≤ 140Å	123.27	149.57
**Matches**	**100%**	**66.7%**
**Molecular properties Varma's rules**	**Limonoids**	
	**swietenolide**	**2-hydroxy-3-*O*-tigloylswietenolide**
HBD[a] + HBA[b] ≤ 9	10	12
MW[c] ≤500 g/mol	486.56	584.66
TPSA[h] ≤ 125Å	123.27	149.57
Log D[i] -2≤x≤5	1.679	1.674
Nrotb[g] ≤ 12	4	7
**Matches**	**80%**	**40%**
**Molecular properties Oprea's rules.**	**Limonoids**	
	**swietenolide**	**2-hydroxy-3-*O*-tigloylswietenolide**
NR[j] ≥3	5	5
Nrotb[g] ≥6	4	7
NRigidB[k] ≥18	35	39
**Matches**	**66.7%**	**100%**

a) HBD= hydrogen bond donors; b) HBA= hydrogen bond acceptors; c) MW= molecular weight; d) Log P= partition coefficient; e) MR= molar refractivity; f) TNA= total number of atoms; g) Nrotb= number of rotatable bonds; h) TPSA= total polar surface area; i) Log D= distribution coefficient; j) NR= number of rings; k) NRigB= number of rigid bonds.

Table S2. Calculated physico-chemical properties of natural degraded limonoids and the degraded limonoid analogs 1-6 by ADMETlab.

Molecular properties Lipinski's rules	Degraded limonoids					
	dictamdiol	calodendrolide	fraxinellone	8,14-epoxyfraxinellone	melazolide A	
HBD[a] ≤ 5	2	0	0	0	2	
HBA[b] ≤ 10	5	4	4	4	4	
MW[c] ≤ 500 g/mol	278.304	260.289	246.262	248.278	212.245	
Log P[d] ≤ 5	1.716	2.762	2.563	2.595	0.238	
**Matches**	100%	100%	100%	100%	100%	
**Molecular properties Lipinski's rules**	**Model compounds**					
	**1**	**2**	**3**	**4**	**5**	**6**
HBD[a] ≤ 5	1	0	1	0	0	0
HBA[b] ≤ 10	3	4	4	4	4	4
MW[c] ≤ 500 g/mol	192.21	234.25	234.25	262.31	290.36	346.47
Log P[d] ≤ 5	1.848	2.419	1.965	3.199	3.980	5.540
**Matches**	100%	100%	100%	100%	100%	75%

Molecular properties Ghose's rules	Degraded limonoids					
	dictamdiol	calodendrolide	fraxinellone	8,14-epoxyfraxinellone	melazolide A	
MW[c] ≤ 500 g/mol	278.304	260.289	246.262	248.278	212.245	
Log P[d] ≤ 5	1.716	2.762	2.563	2.595	0.238	
MR[e] 40<x <130	69.596	66.243	62.589	61.72	52.944	
TNA[f] 20≤x≤70	38	35	32	34	31	
**Matches**	100%	100%	100%	100%	100%	
**Molecular properties Ghose's rules**	**Model compounds**					
	**1**	**2**	**3**	**4**	**5**	**6**
MW[c] ≤ 500 g/mol	192.21	234.25	234.25	262.31	290.36	346.47
Log P[d] ≤ 5	1.848	2.419	1.965	3.199	3.980	5.540
MR[e] 40<x <130	50.54	60.086	58.97	69.32	78.554	97.022
TNA[f] 20≤x≤70	26	31	31	37	43	55
**Matches**	100%	100%	100%	100%	100%	100%

Molecular properties Veber's rules	Degraded limonoids					
	dictamdiol	calodendrolide	fraxinellone	8,14-epoxyfraxinellone	melazolide A	
HBD[a] + HBA[b] ≤ 12	7	4	4	4	6	
Nrotb[g] ≤ 10	1	1	1	1	1	
TPSA[h] ≤ 140Å	79.9	51.97	56.51	51.97	66.76	
**Matches**	100%	100%	100%	100%	100%	
**Molecular properties Veber's rules**	**Model compounds**					
	**1**	**2**	**3**	**4**	**5**	**6**
HBD[a] + HBA[b] ≤ 12	4	4	5	4	4	4
Nrotb[g] ≤ 10	2	4	1	6	8	12
TPSA[h] ≤ 140Å	50.44	56.51	59.67	56.51	56.51	56.51
**Matches**	100%	100%	100%	100%	100%	66.7%

a) HBD= hydrogen bond donors; b) HBA= hydrogen bond acceptors; c) MW= molecular weight; d) Log P= partition coefficient; e) MR= molar refractivity; f) TNA= total number of atoms; g) Nrotb= number of rotatable bonds; h) TPSA= total polar surface area; i) Log D= distribution coefficient; j) NR= number of rings; k) NRigB= number of rigid bonds.

Table S2. Calculated physico-chemical properties of natural degraded limonoids and the degraded limonoid analogs 1-6 by ADMETlab. (Continuation)

Molecular properties Varma's rules	Degraded limonoids					
	dictamdiol	calodendrolide	fraxinellone	8,14-epoxyfraxinellone	melazolide A	
HBD[a] + HBA[b] ≤ 9	7	4	4	4	6	
MW[c] ≤500 g/mol	278.304	260.289	246.262	248.278	212.245	
TPSA[h] ≤ 125Å	79.9	51.97	56.51	51.97	66.76	
Log D[i] -2≤x≤5	0.978	1.513	1.383	1.374	0.189	
Nrotb[g] ≤ 12	1	1	1	1	1	
**Matches**	100%	100%	100%	100%	100%	
**Molecular properties Varma's rules**	**Model compounds**					
	**1**	**2**	**3**	**4**	**5**	**6**
HBD[a] + HBA[b] ≤ 9	4	4	5	4	4	4
MW[c] ≤500 g/mol	192.21	234.25	234.25	262.31	290.36	346.47
TPSA[h] ≤ 125Å	50.44	56.51	59.67	56.51	56.51	56.51
Log D[i] -2≤x≤5	0.863	1.042	0.648	1.421	1.641	2.151
Nrotb[g] ≤ 12	2	4	1	6	8	12
**Matches**	100%	100%	100%	100%	100%	100%
**Molecular properties Oprea's rules**	**Degraded limonoids**					
	**dictamdiol**	**calodendrolide**	**fraxinellone**	**8,14-epoxyfraxinellone**	**melazolide A**	
NR[j] ≥3	3	4	3	4	2	
Nrotb[g] ≥6	1	1	1	1	1	
NRigidB[k] ≥18	21	21	19	20	15	
**Matches**	66.7%	66.7%	66.7%	66.7%	0%	
**Molecular properties Oprea's rules**	**Model compounds**					
	**1**	**2**	**3**	**4**	**5**	**6**
NR[j] ≥3	2	2	3	2	2	2
Nrotb[g] ≥6	2	4	1	6	8	12
NRigidB[k] ≥18	13	14	18	14	14	14
**Matches**	0%	0%	66.7%	33.3%	33.3%	33.3%

a) HBD= hydrogen bond donors; b) HBA= hydrogen bond acceptors; c) MW= molecular weight; d) Log P= partition coefficient; e) MR= molar refractivity; f) TNA= total number of atoms; g) Nrotb= number of rotatable bonds; h) TPSA= total polar surface area; i) Log D= distribution coefficient; j) NR= number of rings; k) NRigB= number of rigid bonds.

Figures S1. NMR and HRMS Spectra of diastereoisomers (±)-1a and (±)-1b

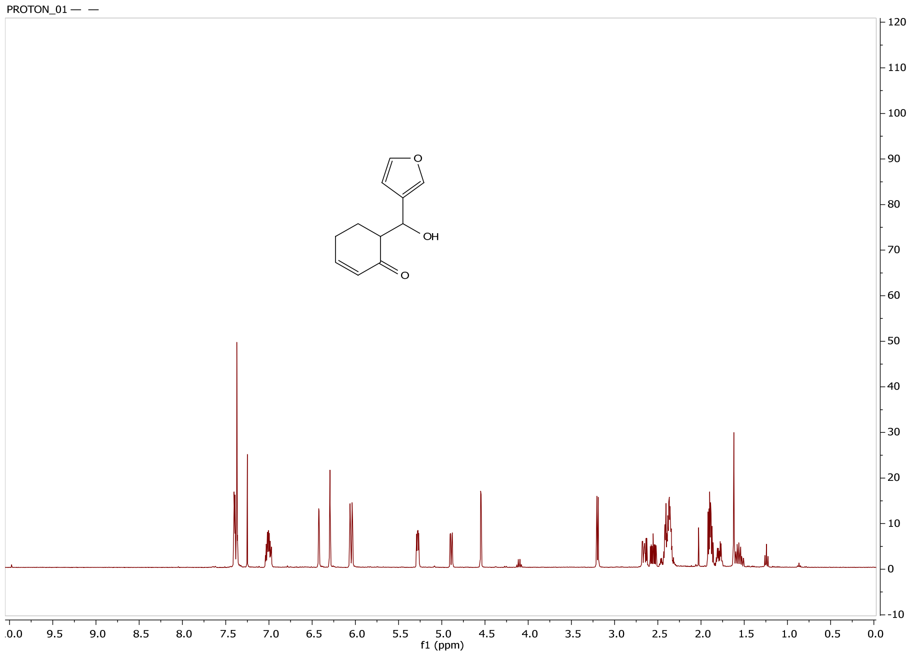

Figure S1.1. ^{1}H NMR spectrum of diastereoisomers (±)-**1a** and (±)-**1b** in $CDCl_3$ (500 MHz).

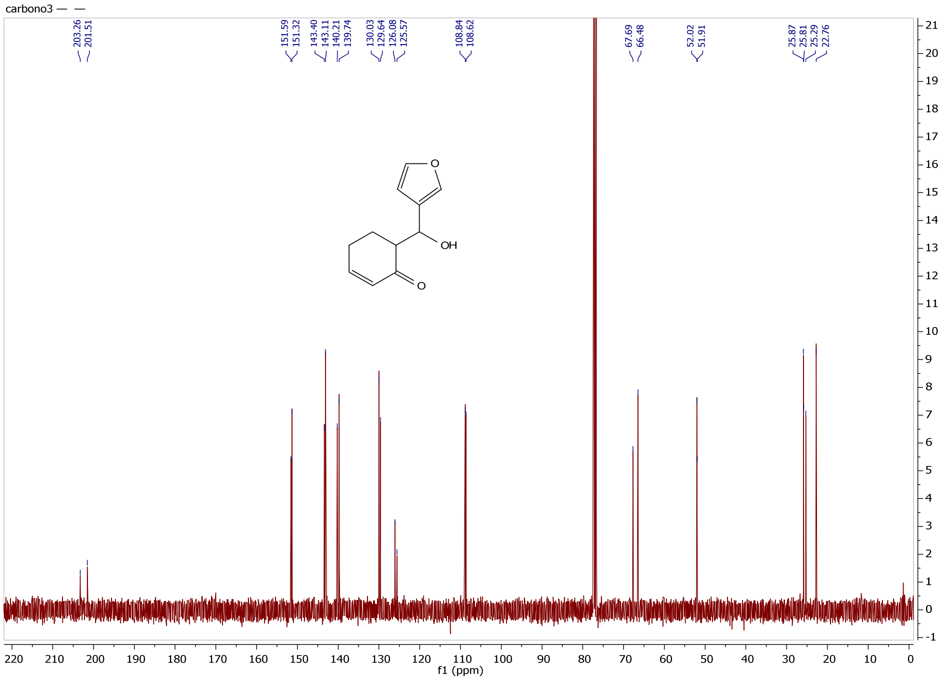

Figure S1.2. ^{13}C NMR spectrum of diastereoisomers (±)-**1a** and (±)-**1b** in $CDCl_3$ (125 MHz).

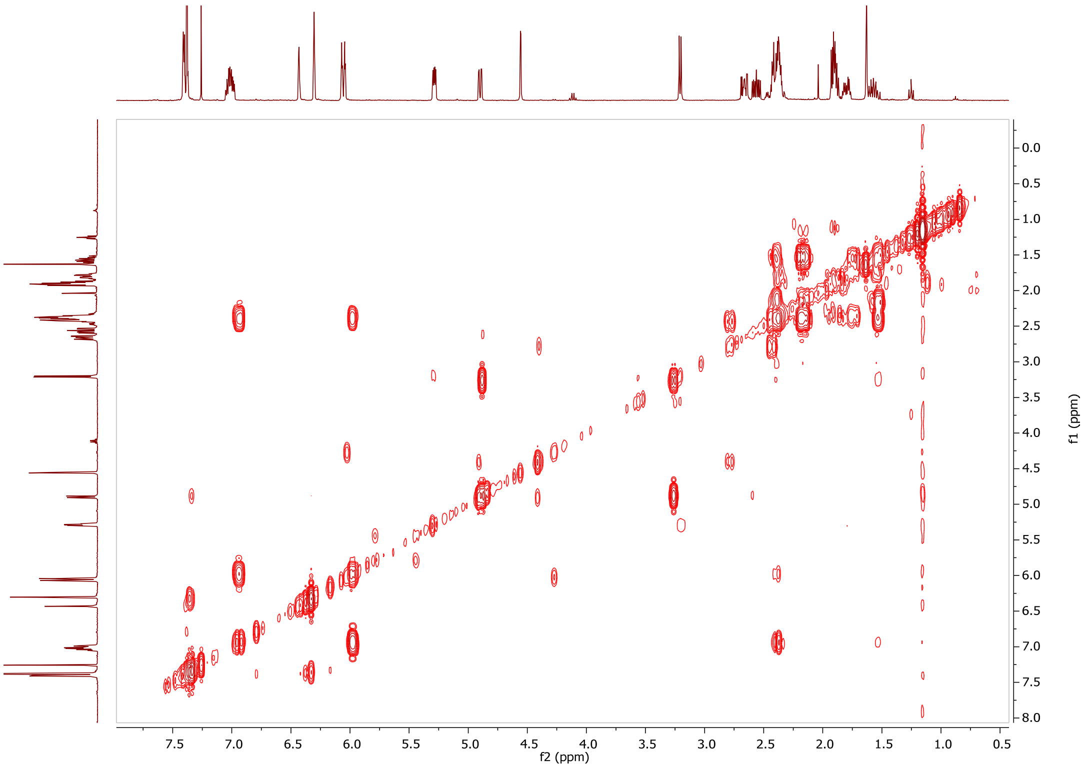

Figure S1.3. g-COSY spectrum of diastereoisomers (±)-**1a** and (±)-**1b** in $CDCl_3$.

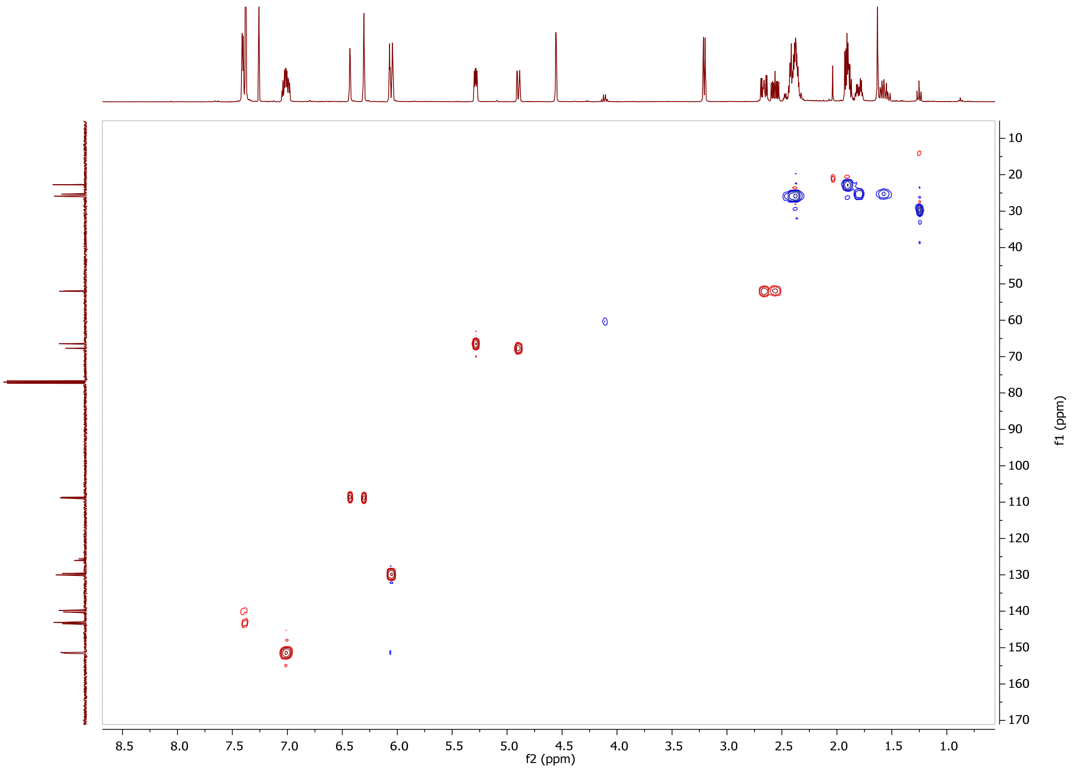

Figure S1.4. HSQC spectrum of diastereoisomers (±)-**1a** and (±)-**1b** in $CDCl_3$.

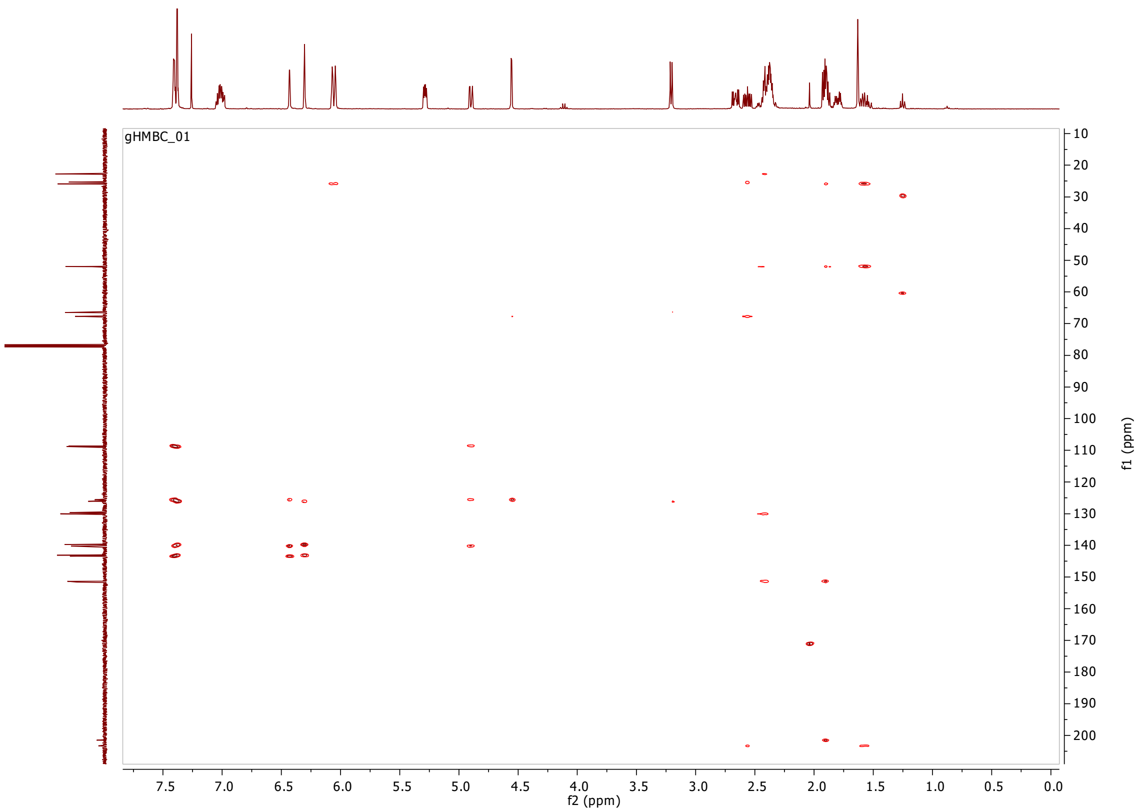

Figure S1.5. HMBC spectrum of diastereoisomers (±)-**1a** and (±)-**1b** in $CDCl_3$.

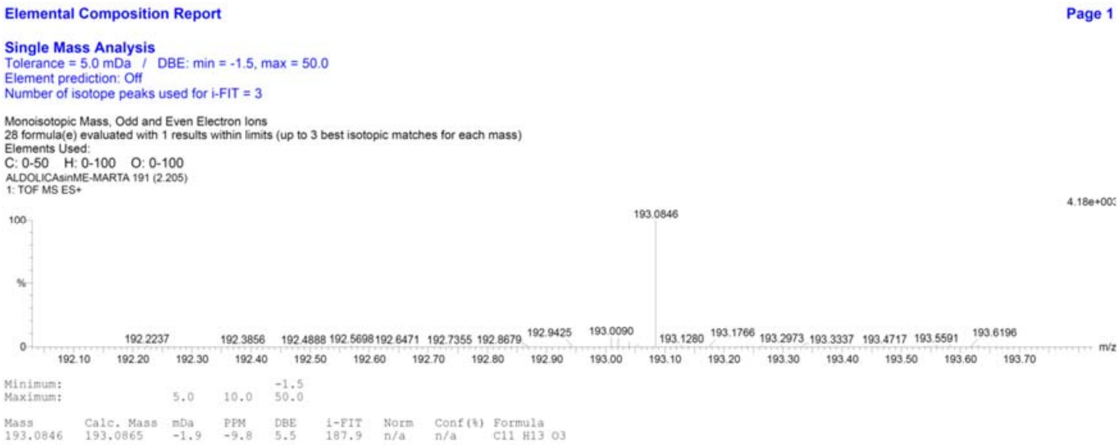

Figure S1.6. HRMS of **1**.

Figures S2. NMR and HRMS Spectra of diastereoisomers (±)-2a and (±)-2b

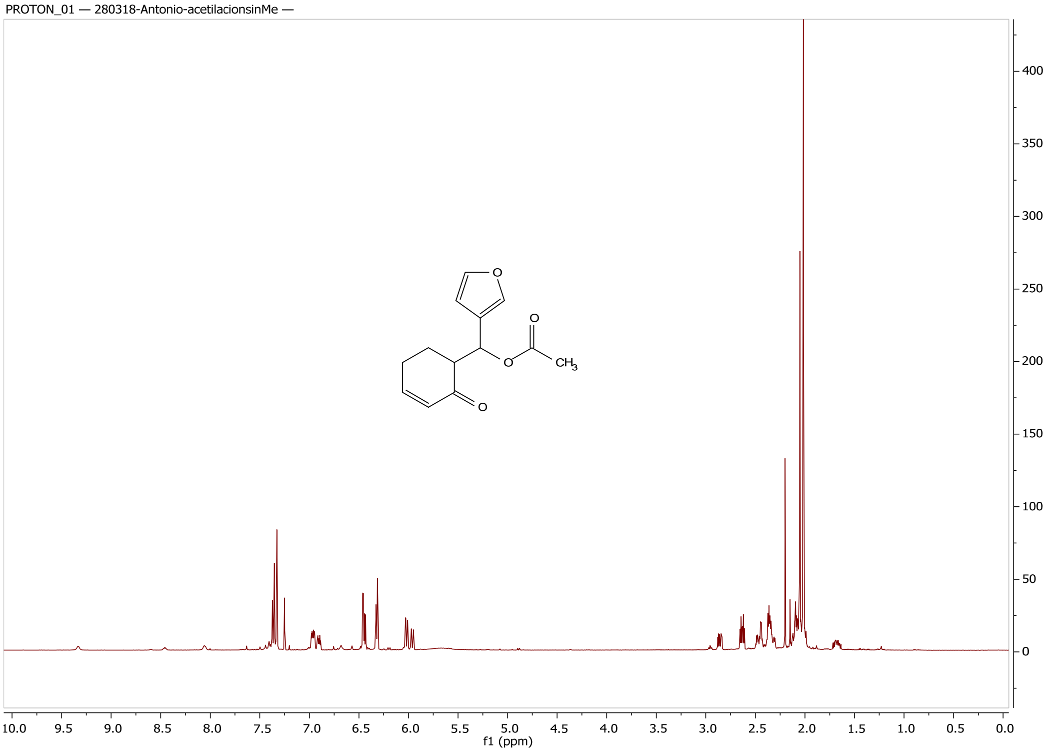

Figure S2.1. ^{1}H NMR spectrum of diastereoisomers (±)-**2a** and (±)-**2b** in $CDCl_3$ (500 MHz).

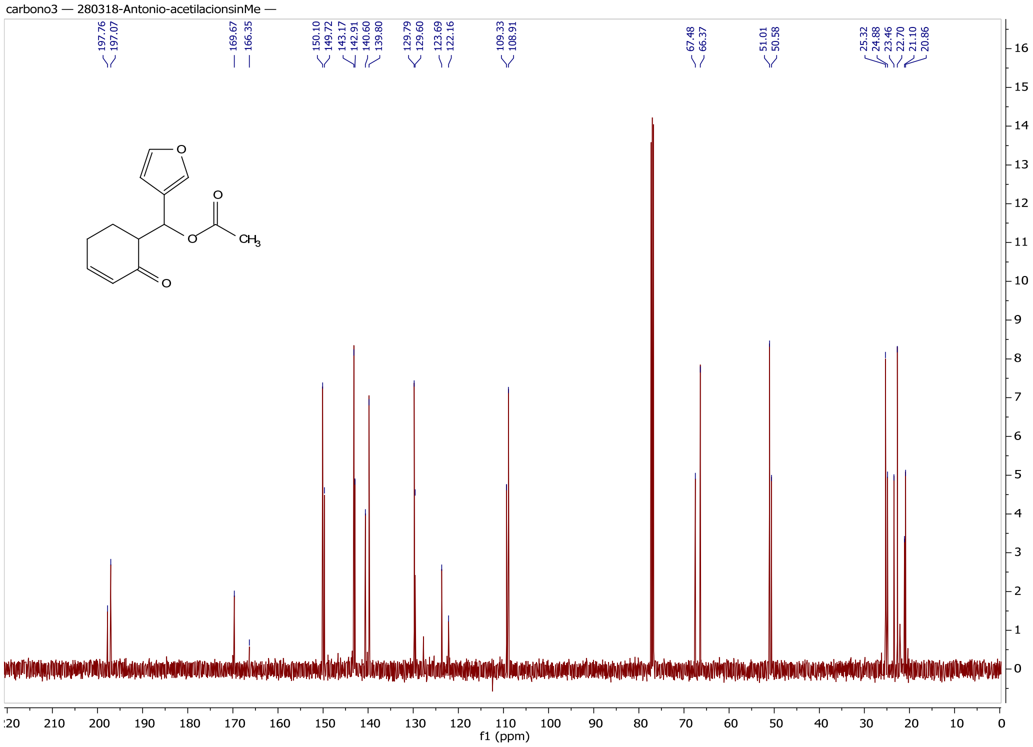

Figure S2.2. ^{13}C NMR spectrum of diastereoisomers (±)-**2a** and (±)-**2b** in $CDCl_3$ (125 MHz).

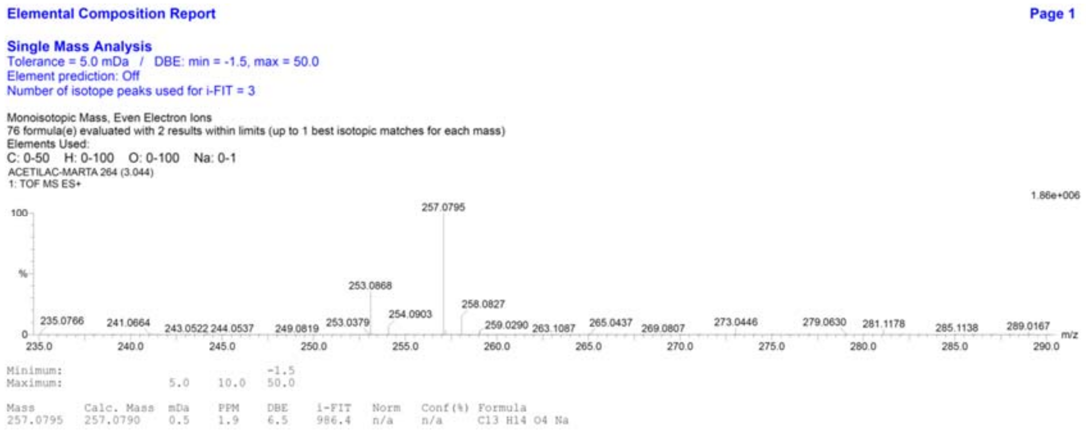

Figure S2.3. HRMS of **2**.

Figures S3. NMR and HRMS Spectra of (±)-3a

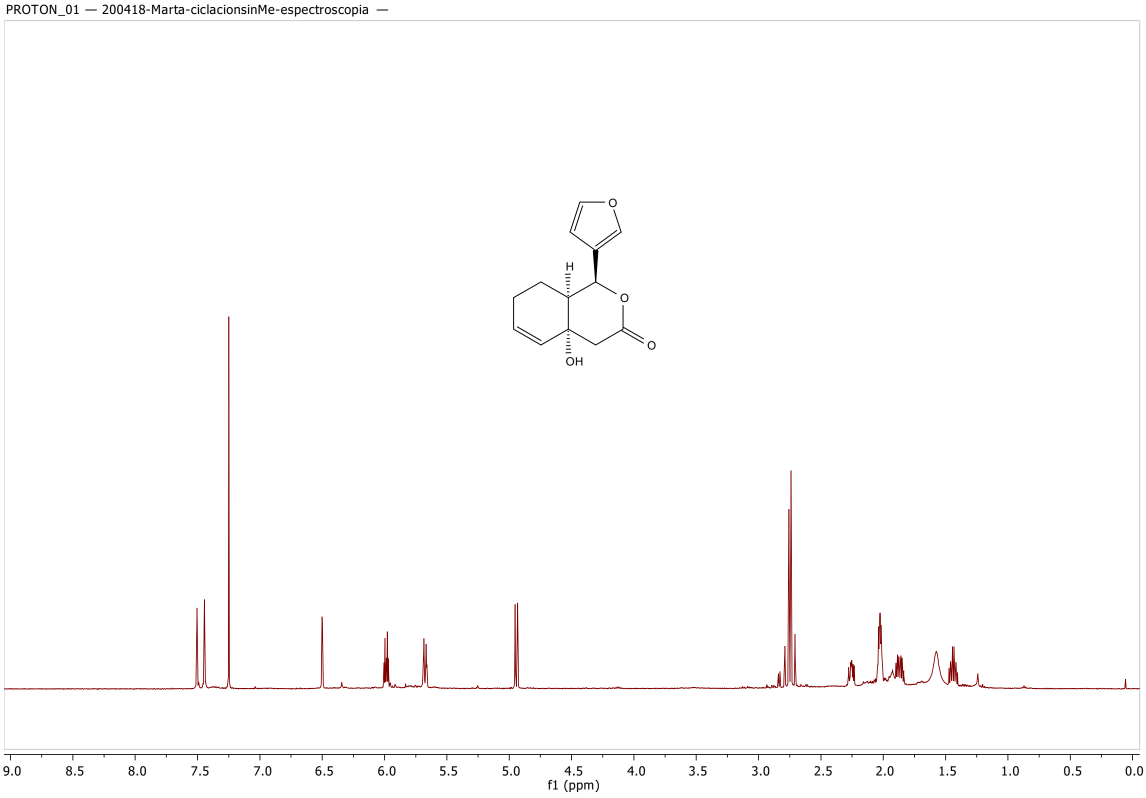

Figure S3.1. ^{1}H NMR spectrum of (±)-**3a** in $CDCl_3$ (500 MHz).

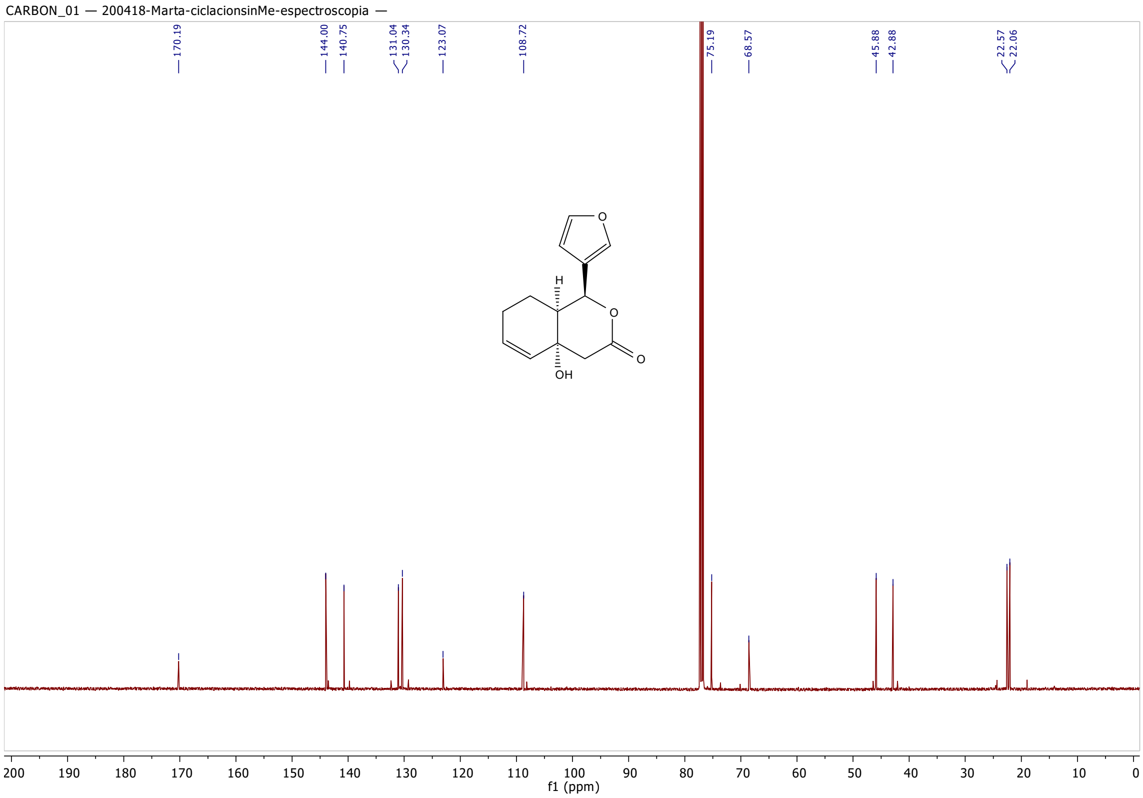

Figure S3.2. ^{13}C NMR spectrum of (±)-**3a** in $CDCl_3$ (125 MHz).

Elemental Composition Report **Page 1**

Single Mass Analysis
Tolerance = 5.0 mDa / DBE: min = -1.5, max = 50.0
Element prediction: Off
Number of isotope peaks used for i-FIT = 3

Monoisotopic Mass, Even Electron Ions
76 formula(e) evaluated with 2 results within limits (up to 5 best isotopic matches for each mass)
Elements Used:
C: 0-100 H: 0-100 O: 0-100 Na: 0-1
CICLADSINME-MARTA 139 (1.614)
1: TOF MS ES+

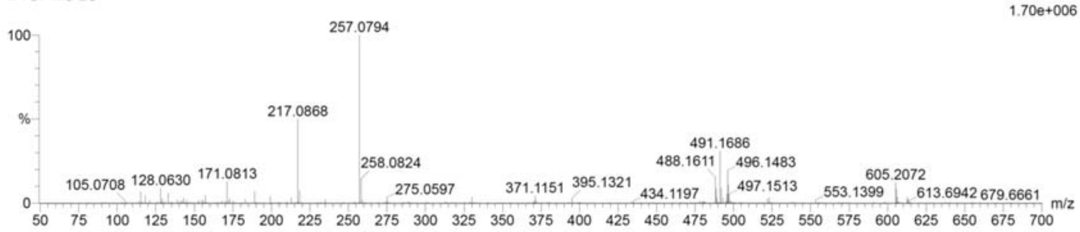

Minimum:				-1.5				
Maximum:		5.0	10.0	50.0				
Mass	Calc. Mass	mDa	PPM	DBE	i-FIT	Norm	Conf(%)	Formula
257.0794	257.0790	0.4	1.6	6.5	933.9	0.000	99.99	C13 H14 O4 Na
	257.0814	-2.0	-7.8	9.5	942.8	8.882	0.01	C15 H13 O4

Figure S3.3. HRMS of (±)-**3a**.

Figures S4. NMR Spectra of diastereoisomers (±)-4a and (±)-4b

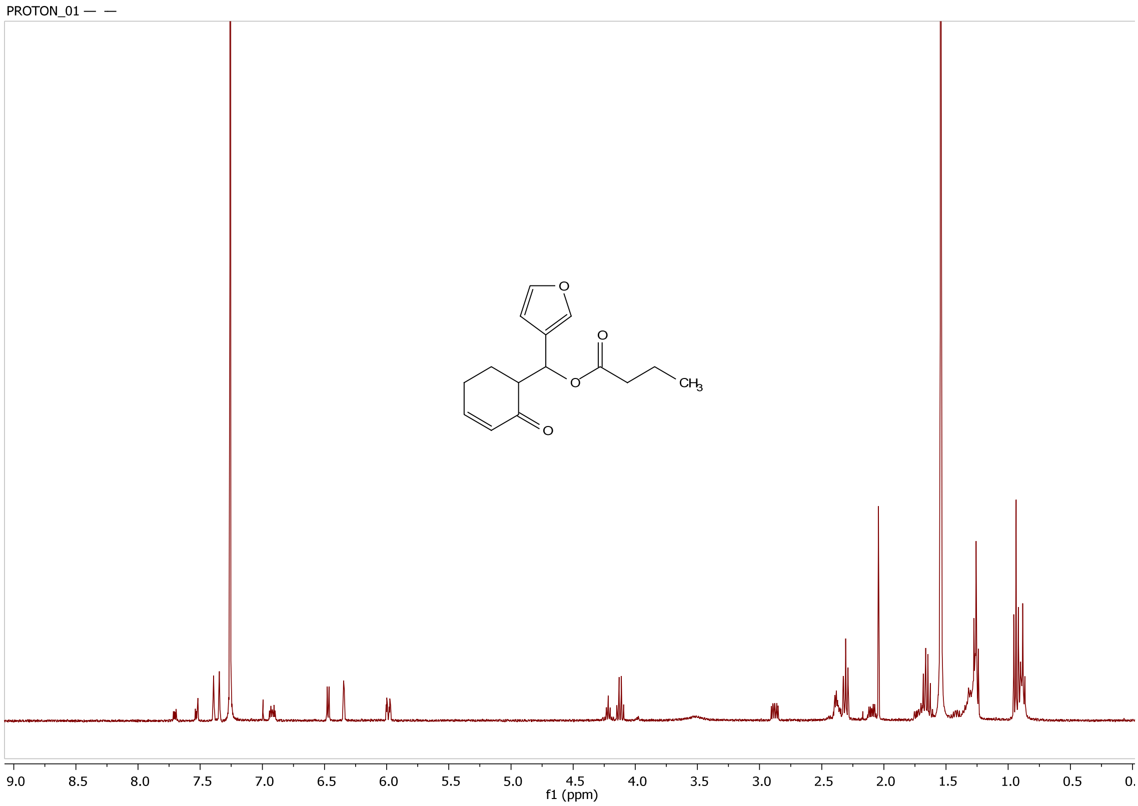

Figure S4.1. ^{1}H NMR spectrum of (±)-**4a** in $CDCl_3$ (500 MHz).

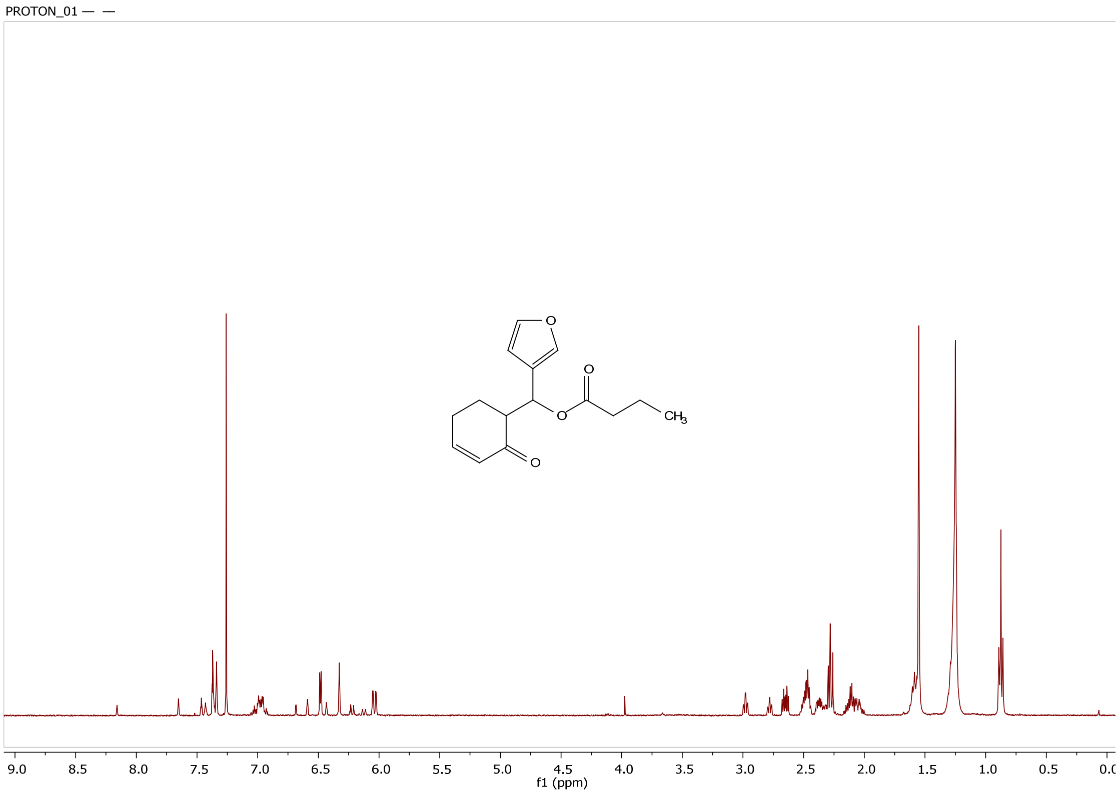

Figure S4.2. ^{1}H NMR spectrum of (±)-**4b** in $CDCl_3$ (500 MHz).

Figures S5. NMR Spectra of diastereoisomers (±)-5a and (±)-5b

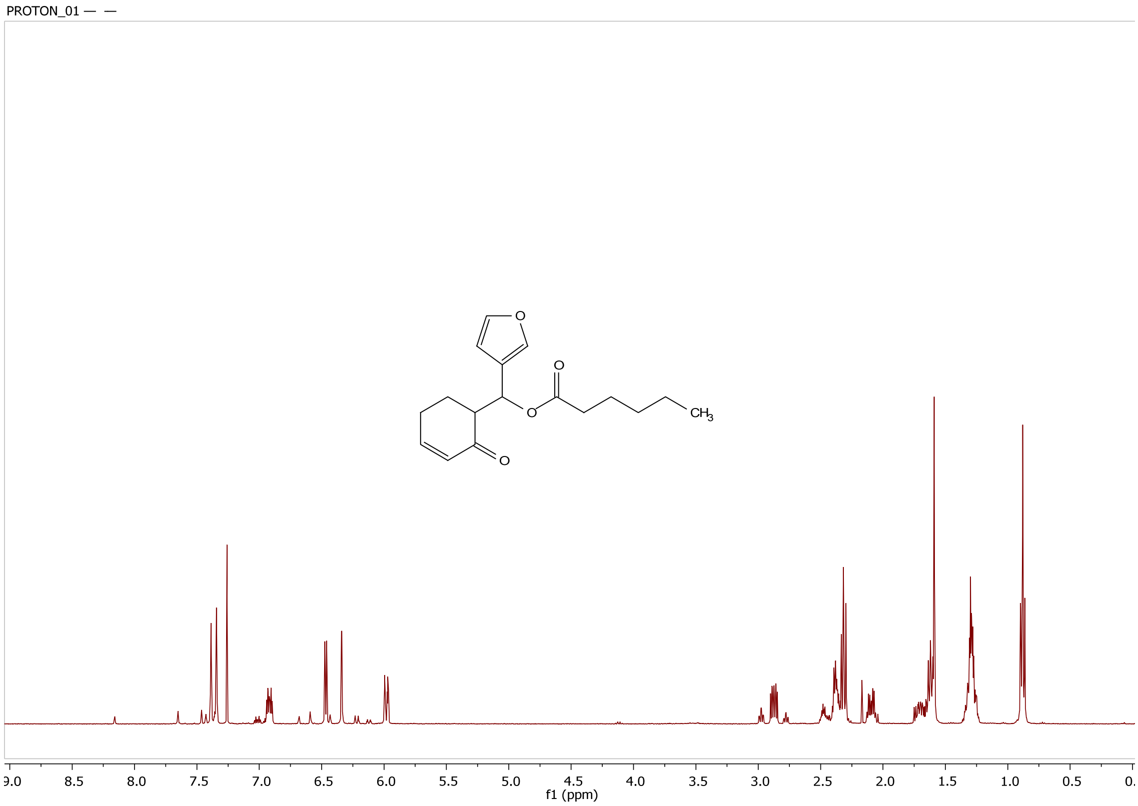

Figure S5.1. ^{1}H NMR spectrum of (±)-**5a** in $CDCl_3$ (500 MHz).

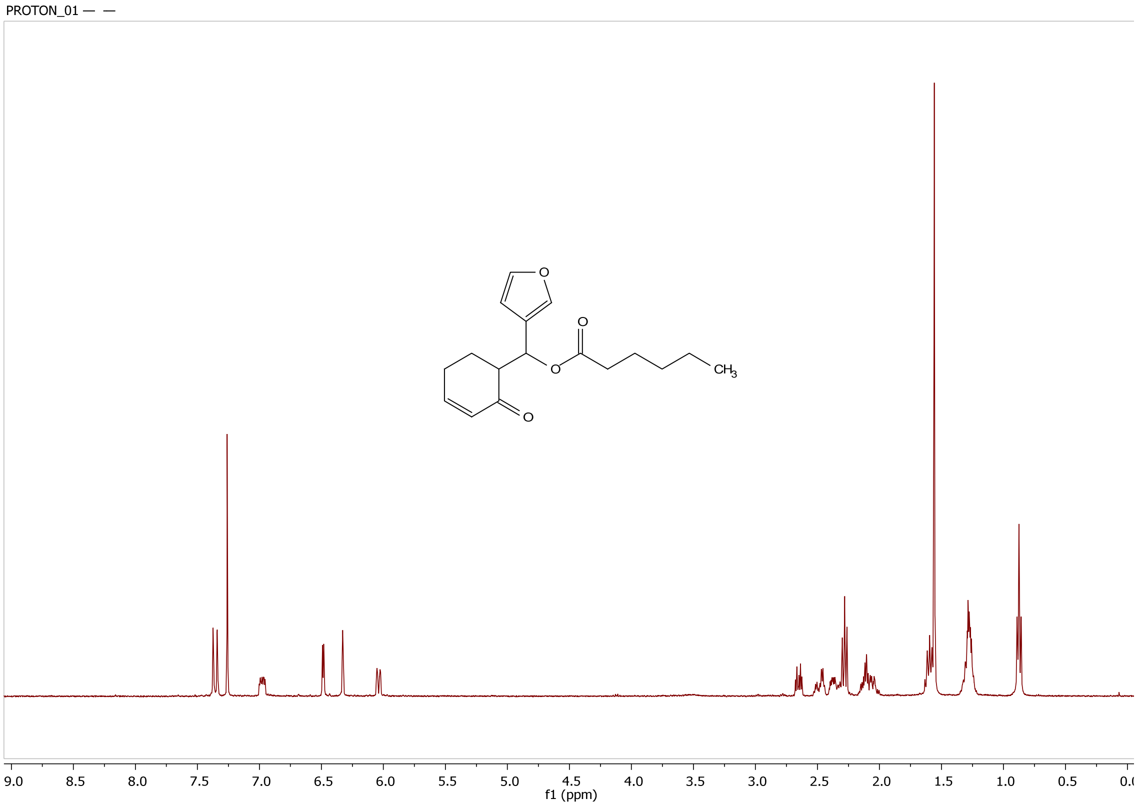

Figure S5.2. ^{1}H NMR spectrum of (±)-**5b** in $CDCl_3$ (500 MHz).

Figures S6. NMR Spectra of diastereoisomers (±)-6a and (±)-6b

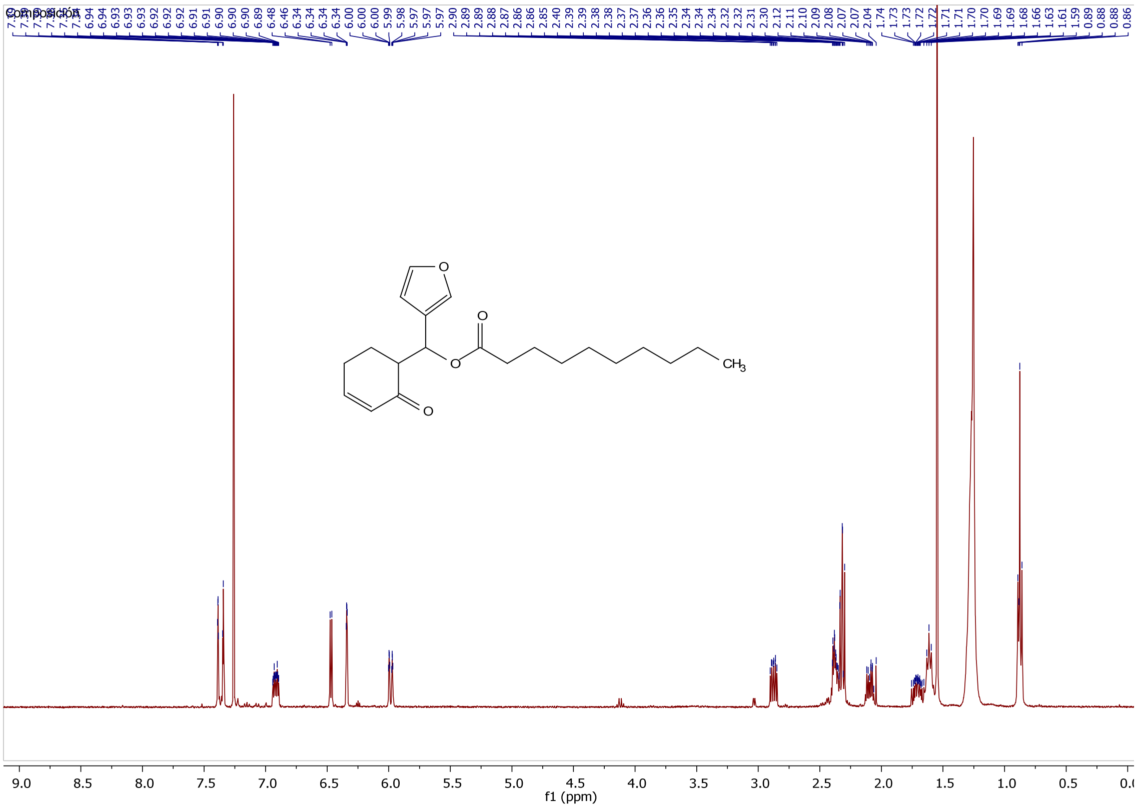

Figure S6.1. ^{1}H NMR spectrum of (±)-**6a** in $CDCl_3$ (500 MHz).

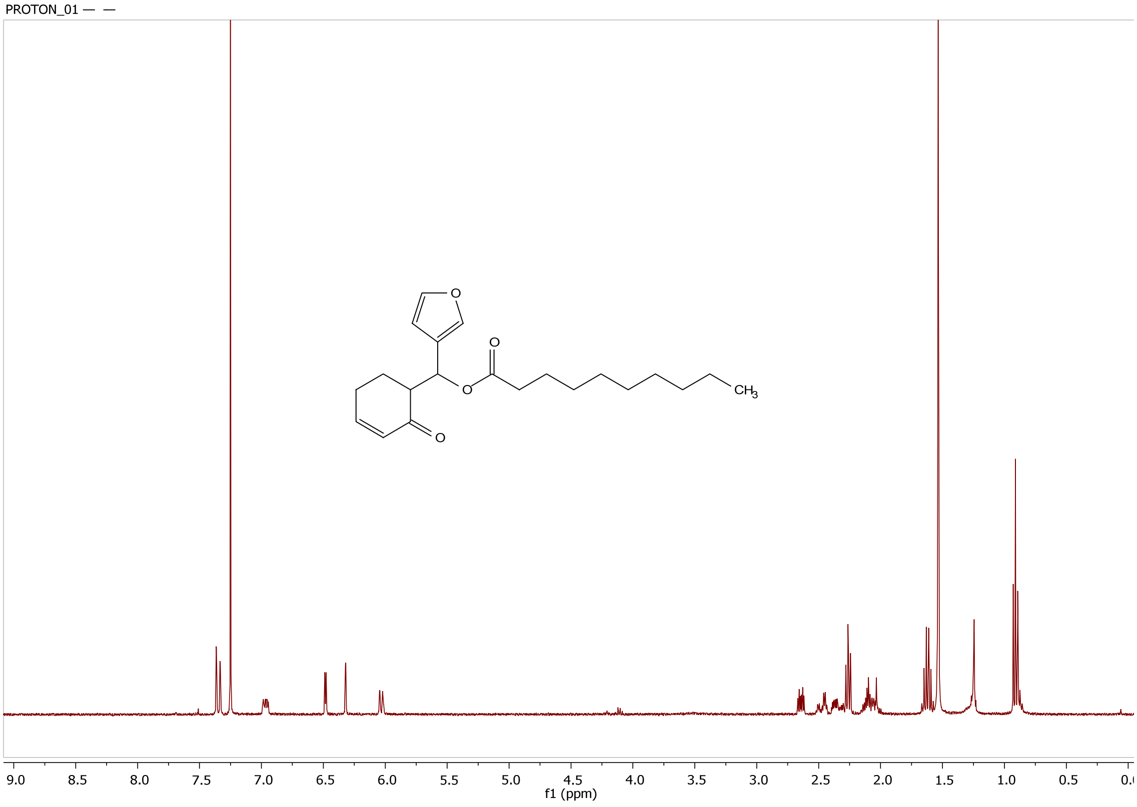

Figure S6.2. ^{1}H NMR spectrum of (±)-**6b** in $CDCl_3$ (500 MHz).

Figures S7. HRMS Spectra of compounds 4, 5 and 6.

Elemental Composition Report **Page 1**

Single Mass Analysis
Tolerance = 5.0 mDa / DBE: min = -1.5, max = 80.0
Element prediction: Off
Number of isotope peaks used for i-FIT = 3

Monoisotopic Mass, Even Electron Ions
77 formula(e) evaluated with 2 results within limits (up to 5 best isotopic matches for each mass)
Elements Used:
C: 0-100 H: 0-100 O: 0-12 Na: 0-1

BUTANOATO 107 (1.003)
1: TOF MS ES+

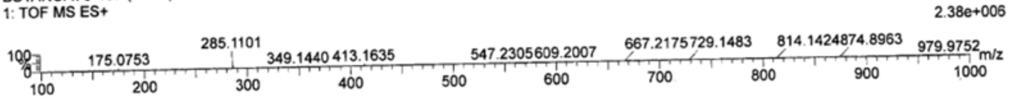

Mass	Calc. Mass	mDa	PPM	DBE	i-FIT	Norm	Conf(%)	Formula
Minimum:				-1.5				
Maximum:		5.0	10.0	80.0				
285.1101	285.1103	-0.2	-0.7	6.5	404.9	0.103	90.26	C15 H18 O4 Na
	285.1127	-2.6	-9.1	9.5	407.1	2.328	9.74	C17 H17 O4

Figure S7.1. HRMS of **4**.

Elemental Composition Report **Page 1**

Single Mass Analysis
Tolerance = 5.0 mDa / DBE: min = -1.5, max = 80.0
Element prediction: Off
Number of isotope peaks used for i-FIT = 3

Monoisotopic Mass, Even Electron Ions
87 formula(e) evaluated with 2 results within limits (up to 5 best isotopic matches for each mass)
Elements Used:
C: 0-100 H: 0-100 O: 0-12 Na: 0-1
HEXANOATO 211 (1.963)
1: TOF MS ES+

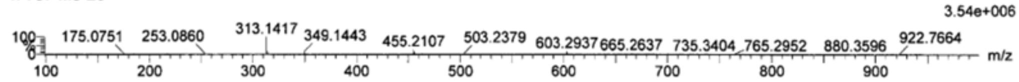

Mass	Calc. Mass	mDa	PPM	DBE	i-FIT	Norm	Conf(%)	Formula
Minimum:				-1.5				
Maximum:		5.0	10.0	80.0				
313.1417	313.1416	0.1	0.3	6.5	510.4	0.135	87.41	C17 H22 O4 Na
	313.1440	-2.3	-7.3	9.5	512.3	2.072	12.59	C19 H21 O4

Figure S7.2. HRMS of **5**.

Elemental Composition Report **Page 1**

Single Mass Analysis
Tolerance = 5.0 mDa / DBE: min = -1.5, max = 80.0
Element prediction: Off
Number of isotope peaks used for i-FIT = 3

Monoisotopic Mass, Even Electron Ions
103 formula(e) evaluated with 2 results within limits (up to 5 best isotopic matches for each mass)
Elements Used:
C: 0-100 H: 0-100 O: 0-12 Na: 0-1
DECANOATO 318 (2.950)
1: TOF MS ES+

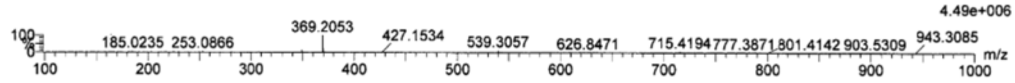

Mass	Calc. Mass	mDa	PPM	DBE	i-FIT	Norm	Conf(%)	Formula
Minimum:				-1.5				
Maximum:		5.0	10.0	80.0				
369.2053	369.2042	1.1	3.0	6.5	620.6	0.085	91.81	C21 H30 O4 Na
	369.2066	-1.3	-3.5	9.5	623.0	2.502	8.19	C23 H29 O4

Figure S7.3. HRMS of **6**.
